# Multi-omics landscapes of colorectal cancer subtypes discriminated by an individualized prognostic signature for 5-fluorouracil-based chemotherapy

**DOI:** 10.1038/oncsis.2016.51

**Published:** 2016-07-18

**Authors:** M Tong, W Zheng, H Li, X Li, L Ao, Y Shen, Q Liang, J Li, G Hong, H Yan, H Cai, M Li, Q Guan, Z Guo

**Affiliations:** 1 Department of Bioinformatics, Key Laboratory of Ministry of Education for Gastrointestinal Cancer, Fujian Medical University, Fuzhou, China

## Abstract

Until recently, few prognostic signatures for colorectal cancer (CRC) patients receiving 5-fluorouracil (5-FU)-based chemotherapy could be used in clinical practice. Here, using transcriptional profiles for a panel of cancer cell lines and three cohorts of CRC patients, we developed a prognostic signature based on within-sample relative expression orderings (REOs) of six gene pairs for stage II–III CRC patients receiving 5-FU-based chemotherapy. This REO-based signature had the unique advantage of being insensitive to experimental batch effects and free of the impractical data normalization requirement. After stratifying 184 CRC samples with multi-omics data from The Cancer Genome Atlas into two prognostic groups using the REO-based signature, we further revealed that patients with high recurrence risk were characterized by frequent gene copy number aberrations reducing 5-FU efficacy and DNA methylation aberrations inducing distinct transcriptional alternations to confer 5-FU resistance. In contrast, patients with low recurrence risk exhibited deficient mismatch repair and carried frequent gene mutations suppressing cell adhesion. These results reveal the multi-omics landscapes determining prognoses of stage II–III CRC patients receiving 5-FU-based chemotherapy.

## Introduction

For all patients with stage III colorectal cancer (CRC) and some patients with stage II CRC likely to be at high risk, 5-fluorouracil (5-FU)-based adjuvant treatments is the first-line treatment.^1, 2^ However, about 20–30% of stage II–III patients receiving 5-FU-based chemotherapy will develop tumor relapse.^1, 3^ Although some molecular markers such as microsatellite instability (MSI) and loss of heterozygosity at chromosome 18q (18qLOH) have been proposed to guide 5-FU-based chemotherapy for CRC patients,^4^ none has been adequately validated for clinical use.^4, 5^ Therefore, it is necessary to explore new prognostic signatures to select patients who most likely to be benefit from the adjuvant chemotherapy after surgery.

Researchers often identified prognostic signatures for chemo-treated patients and then proved its drug benefit predictive value by showing that the identified signatures could not predict prognoses of patients not receiving chemotherapy.^6, 7^ However, this strategy is arguable because patients receiving and not receiving the chemotherapy might have systemic differences in malignant degree of tumor or corporeity.^8^ In order to increase the relevance of prognostic signatures to chemotherapy, some researchers turned to identify prognostic signatures from drug resistant genes extracted from transcriptional profiles for a panel of cancer cell lines.^9, 10, 11^ For example, some studies^9, 11^ extracted drug resistance genes as differentially expressed genes (DEGs) between a particular CRC cell and the corresponding resistant cell induced by 5-FU. However, the majority of such DEGs might represent drug-induced transcriptional changes irrelevant to the drug resistance.^12, 13^ Moreover, a particular cell line model cannot capture the genetic heterogeneity among tumors.^14, 15^ To capture the heterogeneity of cancer in drug response, it would be more reasonable to study a panel of cell lines for each cancer type.^16, 17^ Nevertheless, the clinical relevance of cancer cell models is not guaranteed.^16, 17^ Thus, for candidate signature extracted from cell models, it is necessary to evaluate their clinical relevance before using them to extract drug prognostic signatures.

Notably, current cancer therapeutics is generally dosed in combination,^18, 19^ and thus it is difficult to study the clinical mechanisms of drug resistance for a single drug in clinical practices. Thus, using cell models would be the only practical choice for identifying resistant signatures for a single drug.^9, 20^ Recently, we have made a strict mathematical derivation to prove that if a list of genes represent true resistance genes for a single drug, then their overlaps with clinically relevant drug resistance genes (CRGs) for a combination chemotherapy including this drug should be the CRGs for the shared drug, given that the drugs used in combination had no or limited antagonistic effects.^12^ Here, the CRGs represent the DEGs between the non-responders and responders of patients treated with combination chemotherapy. Thus, if a set of genes associated with 5-FU GI_50_ (50% growth inhibition) of cancer cell lines are significantly consistent with genes correlated with prognoses of CRC patients receiving 5-FU-based combination chemotherapy, then these genes should be CRGs for 5-FU, given that patients with poor or good prognoses should largely represent non-responders or responders to 5-FU treatment. Based on this assumption and in order to increase the relevance of prognostic signatures to a particular drug, for example, 5-FU in this study, we could pre-select 5-FU-resistant genes from cell models, evaluate their clinical relevance and then use these genes to identify prognostic signatures for CRC patients receiving 5-FU-based therapy.

Another problem is that most of the reported transcriptional signatures stratify patients into different risk groups by comparing their risk scores, usually summarized from expression levels of the signature genes, with pre-set risk-score thresholds determined in the training processes.^9, 21, 22, 23^ Owing to experimental batch effects for gene expression profiling,^24^ the applications of such risk-score-based signatures to independent samples require data normalization using a set of samples measured together.^24^ Thus, the risk classification of a sample depends on the heterogeneous risk compositions of the other samples adopted for normalization together.^25, 26^ In contrast, the relative expression orderings (REOs) of genes within a sample are rather robust against to experimental batch effects^27^ and invariable to monotonic data normalization,^25, 28, 29^ rendering them promising for building robust predictors.^25, 30, 31^ Therefore, it is worthwhile to identify REO-based signatures.

In this study, using gene expression profiles of a panel of cancer cell lines with sensitivity data of 5-FU and 200 clinical tissue samples, we identified a REO-based prognostic signature consisting of six gene pairs for stage II–III CRC patients treated with 5-FU-based therapy. The REO-based signature could robustly stratify patients into distinct prognostic groups in two validation data sets. Using the 184 CRC samples from The Cancer Genome Atlas (TCGA) with multi-omics data, we classified the CRC samples into two groups with the same distinct transcriptional characteristics corresponding to the prognostics groups. Instead of analyzing the confounding prognosis data of TCGA with complex therapy regimens which may confound the survival outcome,^32^ we transformed the transcriptional signature to genomic signature and further revealed distinct genomic and epigenetic characteristics of the two CRC groups.

## Results

### Identification of 5-FU-based therapy prognostic gene pair signature

Using 58 NCI-60 cell lines with GI_50_ data for 5-FU derived from nine different tumor types (Supplementary Table S1), we identified 1131 candidate genes whose expression levels were likely to be correlated with GI_50_ values of 5-FU (Spearman correlation, *P*-value <0.05). Then, from these 1131 candidate genes, we extracted 30 genes whose expressions were significantly correlated with the relapse-free survival (RFS) time for 200 CRC patients in stage II–III who underwent 5-FU and folinic acid chemotherapy in the GSE39582 data set (false discovery rate (FDR)<20%, univariate Cox model) (Table 1). Impressively, the concordance score for evaluating the clinical relevance of the 30 genes was 100% (binomial test, *P*-value <1.11E-16, see Materials and methods). Therefore, we defined the 30 genes as clinically relevant 5-FU-resistant genes for further analysis.

**Figure 1:**
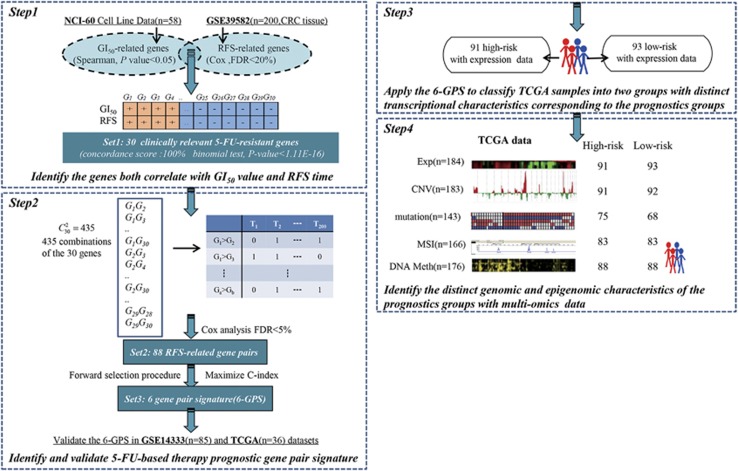
Overview of the workflow used in this study. clinically relevant 5-FU-resistant genes, genes correlates with both cells' GI_50_ value for 5-FU and RFS of CRC patients receiving 5-FU-based chemotherapy; CNV, copy number; Exp, expression; GPS, gene pair signature; Meth, methylation; MSI, microsatellite instability; *n*, the number of samples; +(−), genes positively (negatively) correlated with the GI_50_ values of 5-FU or the RFS of CRC patients receiving 5-FU-based chemotherapy.

**Figure 2:**
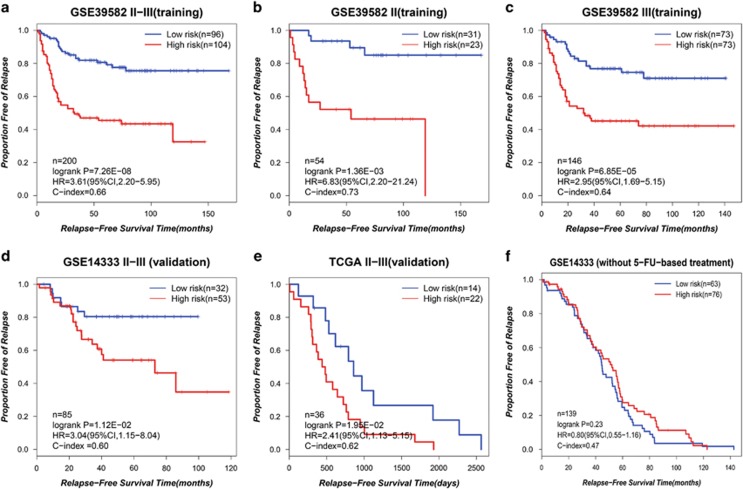
The performance of the 6-GPS for predicting the RFS of the CRC patients. The Kaplan–Meier curves of RFS for the CRC patients treating with 5-FU-based therapy in the training data set (GSE39582) (**a–c**) and the validation data sets ((**d**) GSE14333 and (**e**) TCGA). The Kaplan–Meier curves of RFS for (**f**) the CRC patients without 5-FU-based treatment. *n*, the number of samples.

Then, we developed the prognostic gene pair signature for 5-FU-based therapy according to the flowchart described in Figure 1. For every 2 of the 30 candidate clinically relevant 5-FU-resistant genes, using the GSE39582 data set as the training data, we extracted 88 gene pairs whose REOs were significantly associated with patients' RFS (FDR<5%, univariate Cox model). From these 88 gene pairs, a forward selection procedure was performed to search a set of gene pairs that achieved the highest C-index according to the classification rule as follows: a sample was determined to be at high risk if at least a half of the REOs of the set of gene pairs within this sample voted for high risk; otherwise, the low risk (see Materials and methods). Finally, we obtained six gene pairs consisting of 11 genes, denoted as 6-GPS (Table 2). With the 6-GPS, 104 and 96 of the 200 samples of the training data were stratified into high- and low-risk groups with significantly different RFS time (C-index=0.66; HR=3.61; 95% CI: 2.12–6.03; *P*-value=7.26E-08; Figure 2a). A multivariate Cox analysis for the 200 CRC showed that the 6-GPS remained significantly associated with patients' RFS (HR=3.05; 95% CI: 1.66–5.60; *P*-value=3.36E-04; Table 3), after adjusting for tumor stage, gender, age, tumor location, mismatch repair status and gene mutation (*BRAF* and *KRAS*). Especially, the 6-GPS could successfully stratify the 54 stage II and 146 stage III patients into high- and low-risk groups separately (C-index=0.73; HR=6.83; 95% CI: 2.20–21.24; *P*-value=1.36E-03 for stage II, Figures 2b; C-index=0.64; HR=2.95; 95% CI: 1.69-5.15; *P*-value=6.85E-05 for stage III, Figure 2c).

Then, from the GSE14333 data set, we chose the 85 samples of patients in the stage II–III who underwent 5-FU-based chemotherapy as the first validation data (Table 1). The 6-GPS successfully stratified the 85 patients into a high-risk group with 47 patients and a low-risk group with 38 patients (C-index=0.60; HR=2.64; 95% CI: 1.11–6.24; *P*-value=1.12E-02; Figure 2d). In the second validation data set derived from the TCGA data, which included samples for 36 stage II–III CRC patients with completed RFS after 5-FU-based therapy, 22 and 14 samples were successfully stratified into the high- and low-risk groups with significantly different RFS time(C-index=0.62; HR=2.41; 95% CI: 1.13–5.15; *P*-value=1.95E-02; Figure 2e). The means of the pairwise rank differences of the six gene pairs were 8797, 8954 and 6236 in the GSE39582, GSE14333 and TCGA data sets, respectively. Obviously, a REO-based signature of gene pairs with large pairwise rank differences, which should be difficult to be reversed due to probe detection biases, could be robust against detection biases of different platforms.

**Figure 3:**
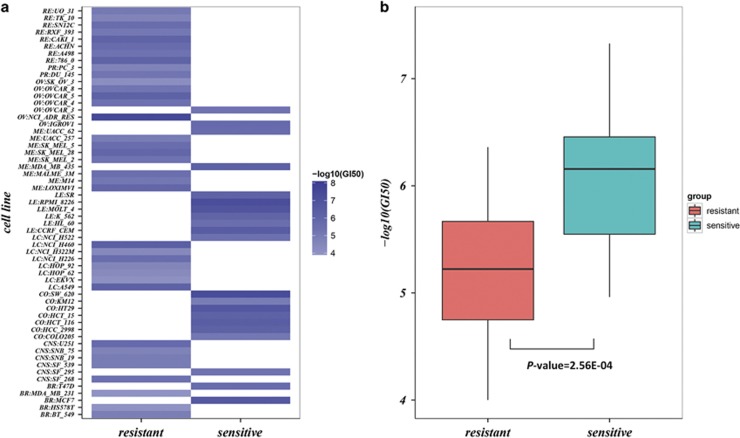
The performance of the 6-GPS for predicting 5-FU resistance for 58 cancer cell lines from the NCI-60 data. The heat map (**a**) and box plot (**b**) of −log_10_ GI_50_ values of the 58 cell lines identified by the 6-GPS. Abbreviations: BR, breast; CNS, central nervous system; CO, colon; LC, non-small cell lung; LE, leukemia; ME, melanoma; OV, ovarian; PR, prostate; RE, renal.

In addition, when using the 6-GPS to analyze the NCI-60 58 cell lines, 37 and 21 were classified as resistant-sensitive cell lines, respectively. The mean of the GI_50_ values in the resistant cells were significantly higher than that in the sensitive cells (Figure 3, Wilcoxon rank-sum test, *P*-value=2.56E-04).

Finally, we used the 6-GPS to stratify the 139 samples from the GSE14333 data set for CRC patients who did not accept 5-FU-based treatment. The result showed that the two groups stratified by the 6-GPS had no significantly different RFS (*P*-value=0.23; Figure 2f), suggesting that the signature was not just prognostic for CRC patients in general but predictive for patients' benefit from 5-FU-based chemotherapy.

### Distinct transcriptional characteristics of the prognostic groups

The validation data sets lacked the necessary clinical data for multivariate Cox analysis. Alternatively, we proved that the transcriptome difference between the prognostic groups identified by the 6-GPS in the validation data sets were consistent with the corresponding difference in the training data set. Using the Wilcoxon rank-sum test, with FDR<5%, we found 7518 DEGs between the high- and low-risk groups stratified from the training data set GSE39582. In the first validation data set GSE14333, 3276 DEGs were found between the high- and low-risk groups (FDR<5%). The two lists of DEGs had 2302 overlapped genes and the concordance score of these genes in the two data sets was 99.22% (binomial test, *P*-value<1.11E-16). Similarly, for the second validation data set with TCGA samples, 708 DEGs were found between the two prognostic groups (FDR<5%). This list of DEGs had 548 and 304 overlapped genes with the corresponding DEGs extracted from the GSE39582 and GSE14333 data sets, with the concordance scores as high as 99.45% (binomial test, *P*-value<1.11E-16) and 99.01% (binomial test, *P*-value<1.11E-16), respectively. These results suggested that differential expressions between the two risk groups classified by the 6-GPS were consistent across independent data sets.

Notably, besides the 36 samples with completed RFS after 5-FU-based therapy, there were other 148 stage II–III CRC samples with gene expression data documented in TCGA. The RFS end points of these samples were unavailable and the overall survival confounded with complex therapy regimens and treatment cycles,^32^ which was unsuitable for prognostic analyses. Nevertheless, we could predict all the 184 samples into 91 high-risk and 93 low-risk patients, respectively. Using the Wilcoxon rank-sum test, we detected 9039 DEGs (FDR<5%) between the two groups, which were significantly consistent with the corresponding DEGs extracted from the GSE39582 and GSE14333 data sets, with the concordance scores as high as 96.94% (binomial test, *P*-value<1.11E-16) and 96.86% (binomial test, *P*-value<1.11E-16), respectively. Based on the reproducibility, we could further classify the CRC samples without completed RFS in TCGA into two groups with the same distinct transcriptional characteristics corresponding to the prognostics groups. Thus, we used all the 184 samples for further analysis. This strategy enabled us to exploit the TCGA multi-omics data to reveal the genomic and epigenetic landscapes of the prognostic groups.

The 2588 DEGs between the prognostic groups, consistently extracted from the GSE39582, GSE14333 and TCGA data sets (Wilcoxon rank-sum test, FDR<10%), were significantly enriched in 36 KEGG pathways (FDR<5%, hypergeometric test, Supplementary Table S2). Out of these pathways, 12 pathways have been reported to be associated with 5-FU sensitivity, including the pyrimidine metabolism pathway promoting 5-FU catabolism,^33^ cell cycle and DNA repair pathways associated with the activity of 5-FU^34, 35^ (Supplementary Table S2).

### Genomic characteristics of the prognostic groups

For the 184 stage II–III TCGA tumors with mRNA-seq profiles, 183, 143 and 166 samples had copy number alteration, somatic mutation data and MSI information data, respectively. This allowed us to further characterize the two prognostic groups with genomic profiles. Notably, we should emphasize that the multi-omics analysis was not used to validate the predictive value but reveal the potential molecular mechanisms determining prognoses of CRC patients treated with 5-FU-based chemotherapy.

**Figure 4:**
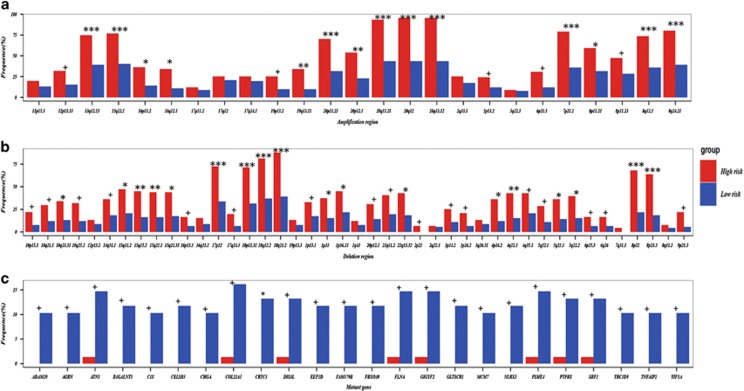
The copy number aberration regions and mutation genes characterizing the high- and low-risk patients, respectively. The frequencies of (**a**) 23 amplification regions, (**b**) 45 deletion regions and (**c**) 24 mutation genes in the high- and low-risk groups, respectively. The 24 mutation genes exhibited significantly higher frequencies in the low-risk group compared with the high-risk group (Fisher test, *P*-value<0.01). ***P-value<0.00001, **P-value<0.0001, *P-value<0.001, ^+^*P*-value<0.05.

Among the 68 chromosome regions with copy number alternation in the 183 CRC samples, we found that 54 (79.41%) chromosome regions had significantly higher variation frequencies in the high-risk group than in the low-risk group (Fisher test, FDR<5%, Figures 4a and b and Supplementary Table S3). Especially, three regions, 20q12, 20q13.12 and 20q11.21, were amplified in almost all the high-risk samples, with the variation frequencies as high as 95.60, 95.60 and 93.41%, while their amplification frequencies in the low-risk group were 43.48, 43.48 and 43.48%, respectively. The deletion frequency of 18q21.2 in the high-risk group was also as high as 87.91% while the frequency was only 39.13% in the low-risk group. It has been reported that CRC patients with 20q13.12 amplification or 18q21.2 deletion still had poor survival after receiving 5-FU-based therapy.^36^ Loss of 18q has been reported to be associated with an adverse response to 5-FU-based adjuvant chemotherapy.^37, 38^

**Figure 5:**
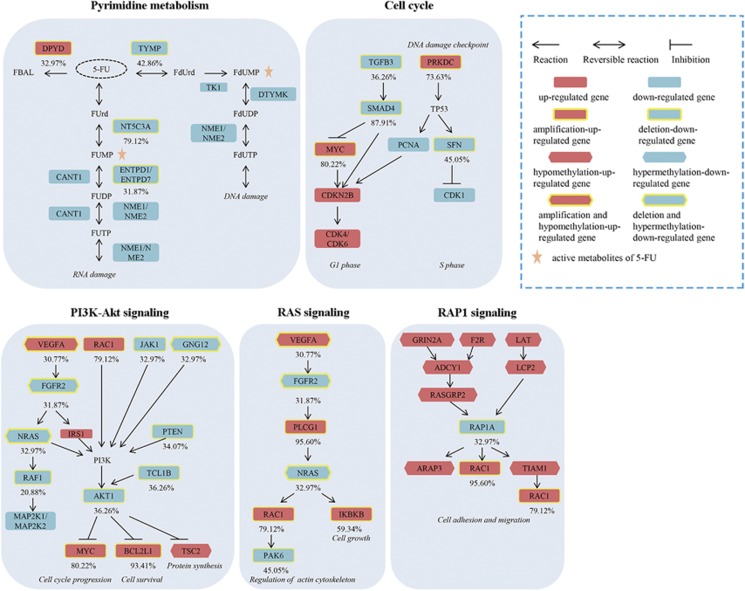
The multi-omic alterations of pathways in the high-risk group. Metabolites of 5-FU: FBAL, a-fluoro-b-alanine; FUrd, fluorouridine; FUMP, fluorouridine monophosphate; FUDP, fluorouridine-5′-diphosphate; FUTP, fluorouridine triphosphate; FdUrd, fluorodeoxyuridine; FdUMP, fluorodeoxyuridine monophosphate; FdUTP, fluorodeoxyuridine triphosphate; FdUDP, fluorodeoxyuridine-5′-diphosphate.

We further analyzed the genomic events that could lead to dysregulations of the 12 pathways associated with 5-FU sensitivity (Supplementary Table S2) and the variation frequency of each pathway among the high-risk samples. To this end, we calculated the prevalence of copy number alterations for each of the pathways in the high-risk samples, representing the number of tumor samples in which at least one gene in the pathway had copy number alternation (Supplementary Data 1). Within the 54 chromosome regions frequently altered in the 91 high-risk samples identified from the TCGA data set, the expression levels of 1179 genes were positively correlated with DNA copy number (Spearman correlation, FDR<5%). In 81.32% of cases, the pyrimidine metabolism pathway included at least one of these 1179 genes, among which *NT5C3A*, *TYMP* and *DPYD* were altered in 79.12, 42.86 and 32.97% of the high-risk samples, respectively. *NT5C3A* and *TYMP* were frequently deleted and underexpressed in the high-risk patients, which might reduce the production of active metabolites of 5-FU, FUMP and FdUMP,^35^ and thus decrease the cytotoxicity of 5-FU (Figure 5). The amplification and overexpression of DPYD, a pyrimidine catabolic enzyme as the initial and rate-limiting factor in 5-FU catabolism, might contribute to poor outcomes of CRC patients receiving 5-FU-based therapy.^39^ In addition, the top three pathways with the most frequent copy number alternations were cell cycle (87.91%), PI3K–Akt signaling pathway (86.81%) and Ras signaling pathway (85.71%) (Figure 5). Many genes with copy number alterations in these pathways can reduce 5-FU efficacy, including *MYC*,^40^ *SMAD4*,^41, 42^ *AKT*,^43^ *BCL2L1*^44^ and *IKBKB*^45^ (Figure 5 and Supplementary Table S4).

**Figure 6:**
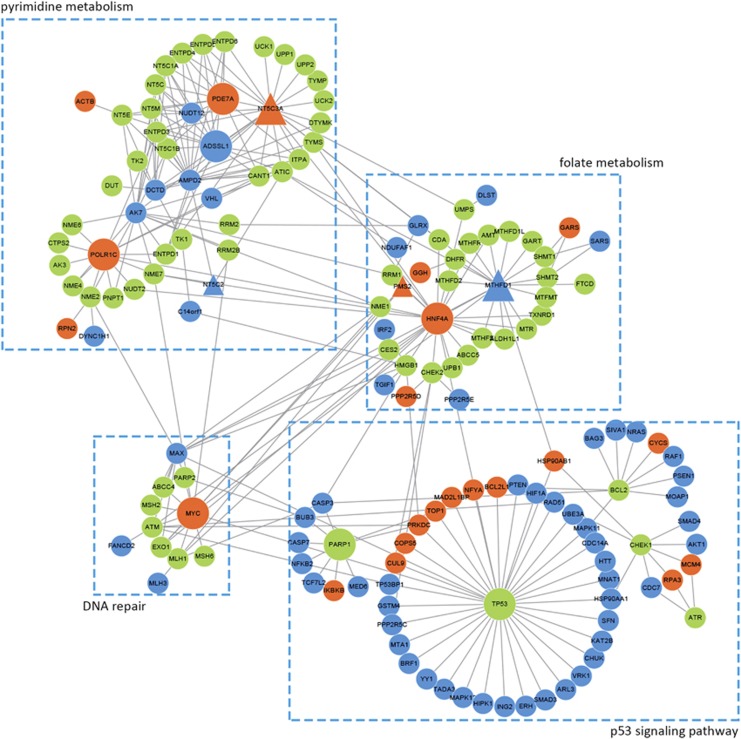
The direct PPI links between the genes with frequent copy number alterations in the high-risk group and 5-FU activity-related genes. 5-FU activity-related genes: genes involved in 5-FU transport, metabolism and other downstream effects (such as DNA repair, apoptosis and cell cycle regulation). The green nodes denoted 5-FU activity-related genes. The red/blue nodes denoted genes with amplification or deletion in the high-risk group. The triangular nodes were the genes overlapped between the genes with frequent copy number alterations and 5-FU activity-related genes.

In addition, of the 1179 genes with frequent copy number alterations in the high-risk group, 853 genes were mapped in the human protein–protein interaction (PPI) network (see Materials and methods). We further analyzed the PPI links between the 1179 and 82 genes involved in 5-FU transport, metabolism and other downstream effects,^34^ denoted as 5-FU activity-related genes. Among the 853 genes with copy number alterations, 10.67% (91) had direct PPI links with at least one of the 82 5-FU activity-related genes, which was significantly higher than the corresponding frequency of 3.90% for the rest of 517 genes without frequent copy number alterations in the high-risk groups (Fisher exact test, *P*-value=3.44E–06). As shown in Figure 6, the hub-nodes mainly involved in pyrimidine metabolism, folate metabolism, p53 signaling pathway^46^ and DNA repair, which were associated with 5-FU activity.

Next, we compared the mutation profiles between the two prognostic groups. Impressively, among the 321 genes that tended to have different mutation frequencies between the two groups (Fisher test, *P*-value<0.05), 320 had higher mutation frequencies in the low-risk group (Supplementary Table S5 and Figure 4c). It was unlikely to be observed by chance (binomial test, *P*-value<1.11E-16). Then, for the 68 low-risk samples, we computed the prevalence of mutations (Supplementary Data 2) in the 12 pathways with transcriptional alternations associated with 5-FU sensitivity (Supplementary Table S2). The top three pathways with the most frequent mutations of these 320 genes were the ECM–receptor interaction (45.59%), focal adhesion (44.12%) and PI3K–Akt signaling pathway (39.71%). Notably, some genes functioning cell adhesion and migration, such as *LAMB1*, *ITGB4* and *ITGA3*, mutated in all the three pathways (Supplementary Table S6). These three genes mutated in 10.29, 11.76 and 5.88% of the low-risk patients, respectively, which might suppress cell adhesion and then decrease the recurrence risk of CRC patients. It has been found that mutations of *LAMB1*,^47^ *ITGB4* and *ITGA3*^48^ were strongly associated with relapse and metastasis in CRC patients.

In addition, the frequency of MSI-High in the low-risk group (27.71%) was also significantly higher than that in the high-risk group (12.05%) (Fisher test, *P*-value=1.87E-02), confirming that stage II–III of CRC patients with MSI-High tumors have a better prognosis compared with patients with MSS/MSI-Low.^49, 50^

### Epigenomic characteristics of the prognostic groups

Among the 9039 DEGs (Wilcoxon rank-sum test, FDR<5%) between the two risk groups in the 176 TCGA samples with DNA methylation profiles, 1555 genes' expression levels were negatively correlated with their methylation levels (Spearman correlation, FDR<5%). The 1555 DEGs were significantly enriched in the PI3K–Akt, cell adhesion molecules and Rap1 signaling pathway (FDR<5%, hypergeometric test; Supplementary Table S7). It has been reported that activation of these pathways could promote cell survival^51, 52^ or inhibit apoptosis^53^ to confer 5-FU resistance. PI3K–Akt and Rap1 signaling pathways also included genes with frequent copy number altered (Figure 5), which suggested that the dysregulation of these pathways might be induced by both genomic and epigenomic alternations.

## Discussion

There is a compelling need to identify CRC patients who will benefit from 5-FU-based adjuvant therapy. In this study, we firstly identified 30 genes whose expression levels correlated with GI_50_ values of 5-FU and successfully validated their clinical relevance to prognoses of CRC patients treated with 5-FU-based therapy. Then, from these 30 genes, we extracted a prognostic signature based on the within-sample ROEs of six gene pairs for stage II–III CRC patients receiving 5-FU-based therapy. Recently, Guinney *et al.*^54^ have created a methodological gold standard for the taxonomy of CRC and reported the gene expression-based consensus molecular subtypes (CMS) of CRC, which include CMS1, CMS2, CMS3 and CMS4. We applied the Single Sample Predictor classifier provided by the authors to classify the CRC patients receiving 5-FU-based therapy. We found that the high-risk patients tended to be classified to CMS4 and the low-risk patients tended to be labeled as CMS2 or CMS3(Supplementary Table S9). The results confirmed that the CMS4 tumors have worse overall survival than the CMS2 and CMS3 tumors.^54^ Notably, the association of CMS subgroups with the chemotherapy efficacy is still unknown. The consensual description of CRC heterogeneity could be used to predefine patient subgroups, from which we could further identify patients that benefit from specific chemotherapy. However, the classifier reported by Guinney *et al* still requires data normalization. Different form the CMS classifier, the REO-based signature, which is largely free of experimental batch effect and does not need data normalization, enables us to distinguish stage II–III CRC patients who are more likely to benefit from 5-FU-based therapy. The comparisons between the study reported by Guinney *et al* and our work was displayed in the Supplementary Table S10.

Notably, it would be more appropriate to filter out gene pairs with unstable ordering in data sets produced by different platforms with different detection biases.^29, 55^ However, except for the GSE39582 and GSE14333 data sets analyzed in this study, no other CRC samples with definite 5-FU-based chemotherapy data produced by other platforms were found. Many data sets of CRC have been misused due to the unclear and incomplete data annotation in public data sources.^56^ Additional clinical data sets are needed to advance research into the robustness of REO-based signature in different platforms. In addition, it can be expected that REOs deduced from transcriptional abundance measured by reverse transcriptase PCR (RT–PCR) tend to be robust against batch effects existing in RT–PCR experiments.^24^ Thus, it is worth developing RT–PCR kit to measure the REOs of the six gene pairs for the clinical application of the REO-based signature. It is also necessary to explore the specific applications of REO-based signature such as evaluating the robustness of the assays in paraffin-embedded specimens.

In summary, the REO-based signature, which is largely free of experimental batch effect and does not need data normalization, could distinguish stage II–III CRC patients who are more likely to benefit from 5-FU-based therapy after surgery. The robustness of the signature enables us to integrate the multi-omics data documented in TCGA to characterize prognostic groups comprehensively.

## Materials and methods

### Data acquisition and processing

Drug-sensitivity data and expression profiling data for the NCI-60 were obtained from the NCI DTP (Table 1). Tissue samples were downloaded from Gene Expression Omnibus (GEO) and TCGA (Table 1). When using the REO-based signature to predict prognoses of patients or sensitivity of cell lines in a one-by-one manner (at the individual level), we just used the robust microarray average (RMA) to perform the background-adjust to reduce the within-sample optical and nonspecific binding noise.^57^ In order to select DEGs between two prognostic groups predicted by the REO-based prognostic signature^57^ and performed the correlation analysis, data sets generated from the Affymetrix platform were pre-processed using the RMA with quantile normalization. Each probe-set ID was mapped to its Entrez gene ID with the corresponding custom CDF files. If multiple probe-sets were mapped to the same gene, the expression value for the gene was defined as the arithmetic mean of the values of the multiple probe-sets (on the log2 scale).

For data sets from TCGA, gene expression data of level 3 derived from Illumina HiSeq 2000 RNA Sequencing Version 2 analysis, somatic mutation data of level 2 derived from Illumina Genome Analyzer DNA Sequencing, methylation data of level 3 derived from Illumina Infinium Human DNA Methylation 450 platform were chosen and downloaded from TCGA portal. Copy number data of level 4 derived from Genome-Wide Human SNP Array 6.0 for TCGA samples analyzed by the GISTIC 2.0 algorithm^58^ were downloaded from Firehose. Using the significant regions of gain or loss identified by GISTIC 2.0, we assigned a discrete copy number alteration status to each gene in each sample. For gene mutation data, only the non-synonymous mutations were included and a discrete mutation profile including 15 044 genes were generated. For DNA methylation profiles, we focused on analyzing the 25 978 CpG sites located at the promoter regions of genes. The SVA package was used to remove batch effects and other unwanted variation.^59^

### Concordance scores

For DEGs from two independent data sets sharing *k* DEGs, of which *s* genes had the same up- or downregulation directions, the concordance score was calculated as *s/k*.

If *k* genes were found to both correlate with the GI_50_ values of 5-FU in cell lines and the RFS of CRC patients treated with 5-FU-based chemotherapy, among which *s* genes had the same signs positively (or negatively) correlated with the GI_50_ values of 5-FU in cell lines and correspondingly negatively (or positively) correlated with the RFS of CRC patients, then the concordance score was calculated as *s/k*.

The probability of observing a concordance score (*s/k*) by chance can be evaluated using the cumulative binomial distribution model as follows:


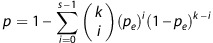


in which *p*_*e*_ is the probability of one gene having the same dysregulation direction in two gene lists by random chance (here, *p*_*e*_=0.5).

### Correlation and survival analysis

The Spearman's rank correlation analysis was used to evaluate the correlation of genes expression levels with GI_50_ values of 5-FU for NCI-60 cell lines. The RFS was defined as the time from the date of initial surgical resection to the date of relapse or last contact. Survival curves were estimated using the Kaplan–Meier method and were compared using the log-rank test. The univariate Cox proportional-hazards regression model was used to evaluate the correlation of gene expression levels and REOs of gene pairs with the RFS of CRC patients. The multivariate Cox proportional-hazards regression model was used to evaluate the independent prognostic value of the signature after adjusting for clinical factors including tumor stage, age, gender tumor location, mismatch repair status and gene mutation (*BRAF* and *KRAS*). *P*-values were adjusted using the Benjamini and Hochberg procedure.^60^

### Identification of 5-FU-based therapy prognostic gene pair signature

All possible pairs were combined between every two candidate genes correlated with both cells' GI_50_ for 5-FU and patients' prognoses. Let *Ga* and *Gb* represent the expression levels of gene a and gene b, respectively. For each gene pair (*Ga* and *Gb*), correlation between a specific REO pattern (*Ga>Gb* or *Ga<Gb*) and RFS was performed by univariate Cox regress analysis. Controlling the FDR at the 5% level, gene pairs having correlation with survival were defined as *Set1*. Then, for a gene pair in *Set1*, if its reversal REO (e.g. *Ga*>*Gb→Ga<Gb*) was associated with worse or better survival, then we considered that the reversal REO in a cancer sample voted for high- or low-risk, whereas the non-reversal REO in a cancer sample voted for low or high risk. According to the REO pattern of gene pairs from *Set1*, a sample was determined to be high risk if at least a half of the REOs of the set of gene pairs within this sample voted for high risk; otherwise, this sample was classified into the low-risk group. C-index values were calculated in the training data set for each gene pair.^61^ All gene pairs were sorted in descending order according to the C-index values, which was defined as *Set2.* Finally, a forward selection procedure was used to search a set of gene pairs that achieved the highest C-index. We chose the gene pair with the highest C-index value from *Set2* as a seed, and added the next gene pair to the signature one at a time until the C-index did not increase. A set of gene pairs with the highest C-index was chosen as *Set3*, which was further defined as the GPS. Figure 1 describes the flowchart.

### Genomic and epigenomic analysis of the prognostic groups

Fisher exact test was used to assess different frequencies of mutation and copy number aberrations between groups. Spearman rank correlation analysis was used to evaluate the correlation between copy number, or methylation level, and expression changes. The Wilcoxon rank-sum test was used to detect DEGs between two groups of samples.

### Human PPI data

The PPI data were downloaded from HPRD,^62^ IntAct,^63^ MIPS,^64^ MINT,^65^ DIP,^66^ BIND,^67^ KEGG^68^ and neighboring reactions.^69^ We compiled an integrated interaction network of 142 583 distinct interactions involving 13 693 human proteins.^70^ The Fisher exact test was used to test whether the direct PPI links between two gene sets were significant more that what expected by random chance.

In the network, 82 genes involved in 5-FU transport, metabolism and other downstream effects, denoted as 5-FU activity-related genes, which were collected from a previous study^34^ (Supplementary Table S8).

### Functional enrichment analysis

The functional categories for enrichment analysis were downloaded from KEGG.^68^ The hypergeometric distribution model was used to test whether a set of genes observed in a functional term was significantly more than what expected by random chance. All statistical analyses were performed using the R 3.12.

## Figures and Tables

**Table 1:** Data sets analyzed in this study

*Data source*	*Data type*	*Platform*	*Stage*	*Treatment*	*Sample size*
*Cell line*					
NCI-60	mRNA	Affymetrix U133 A	—	5-FU	58

*CRC tissue*					
GSE39582	mRNA	Affymetrix U133 Plus 2.0	II–III	5-FU and folinic acid	200
GSE14333^a^	mRNA	Affymetrix U133 Plus 2.0	II–III	5-FU-based	85
GSE14333^a^	mRNA	Affymetrix U133 Plus 2.0	I–III	Without 5-FU-based treatment	139
TCGA	mRNA	IlluminaHiSeq_RNASeqV2	II–III	5-FU-based	184
TCGA^b^	DNA Copy number	Genome-Wide Human SNP Array 6.0	II–III	5-FU-based	183
TCGA^b^	Somatic mutation	Illumina Genome Analyzer DNA Sequencing	II–III	5-FU-based	143
TCGA^b^	MSI	Microsatellite Instability Analysis	II–III	5-FU-based	166
TCGA^b^	DNA methylation	Illumina Infinium Human DNA Methylation 450	II–III	5-FU-based	176

Abbreviations: 5-FU, 5-fluorouracil; MSI, microsatellite instability.

a In this data set, there were 85 samples of patients treated with 5-FU-based chemotherapy and 139 samples of patients did not accept 5-FU-based treatment. These two groups of samples were analyzed.

b Among the 184 TCGA samples with mRNA-seq profiles, 183, 143, 166 and 176 samples also had copy number, somatic mutation, MSI and DNA methylation data.

**Table 2:** Composition of the 6-GPS

*Signature*	*ROEs (*R_*a*_*>*R_*b*_)	β	P*-value*	*FDR*	*C-index*
Gene pair 1	*CHTOP>CAPN2*	1.22	6.37E-05	1.89E-03	0.63
Gene pair 2	*MRPL4>AXL*	0.98	4.12E-05	1.70E-03	0.60
Gene pair 3	*SLC19A1>NREP*	1.09	2.19E-03	1.43E-02	0.59
Gene pair 4	*PUS1>LTBP2*	0.61	8.74E-03	3.39E-02	0.57
Gene pair 5	*MCM2>IFRD2*	0.66	1.18E-02	3.89E-02	0.57
Gene pair 6	*SLC19A1>WWC2*	1.20	9.92E-05	2.41E-03	0.56

Note: ROEs represent the relative expression ordering of gene pair (*R*_a_>*R*_f_); *β* and *P*-value are the statistics calculated from the univariate Cox regression model. *β* represents the risk coefficient of the REO for gene pair (a, b), where *β*>0 indicates that R_a_> R_b_ is a risk factor, otherwise a protective factor; *P*-value represents the significance of the REO for gene pair (a, b). All the *P*-values were adjusted using the Benjamini–Hochberg procedure.

**Table 3:** Univariate and multivariate Cox regression analysis for the GSE39582 data set

*Characteristics*	*Univariate analysis*		*Multivariate analysis*	
	*HR*^a^ *(95% CI)*	P*-value*	*HR (95% CI)*	P*-value*
**6-GPS (low risk(ref)/high risk)**	**3.61 (2.20,5.95)**	**7.26E-08**	**3.05 (1.66,5.60)**	**3.36E-04**
Tumor stage (II(ref)/III)	1.43 (0.83, 2.46)	2.00E-01	1.56 (0.78, 3.10)	2.09E-01
Gender (femal (ref)/male)	1.05 (0.67, 1.66)	8.30E-01	0.74 (0.42, 1.33)	3.16E-01
Age (⩽70 (ref)/>70)	1.03 (0.62, 1.71)	9.00E-01	0.78 (0.40, 1.53)	4.73E-01
Tumor location (proximal (ref)/distal)	1.38 (0.83, 2.29)	2.10E-01	1.84 (0.93, 3.67)	8.16E-02
MMR status (pMMR (ref)/dMMR)	0.80 (0.29, 2.20)	6.70E-01	1.74 (0.49, 6.23)	3.94E-01
*BRAF* mutation (WT (ref)/M)	0.71 (0.17, 2.93)	6.40E-01	0.62 (0.073, 5.25)	6.61E-01
*KRAS* mutation (WT (ref)/M)	1.26 (0.78, 2.04)	3.40E-01	1.35 (0.73, 2.50)	3.44E-01

Abbreviations: dMMR, MMR-deficient; HR, hazard ratio; M, mutation; MMR, mismatch repair; pMMR, MMR-proficient; ref, reference group in calculation of HR; WT, wild-type.

Bold parts indicates the Cox regression analysis results for the 5-FU-based therapy prognostic gene pair signature.
