# Supplementary material for: Multi-omics landscapes of colorectal cancer subtypes discriminated by an individualized prognostic signature for 5-fluorouracil-based chemotherapy
Source: Oncogenesis. 2016 Jul 18;5(7):e242–. doi: 10.1038/oncsis.2016.51 (PMC5399173; doi:10.1038/oncsis.2016.51)
Supplement: Supplementary Information [file oncsis201651x1.docx]

**Multi-omics landscapes of colorectal cancer subtypes discriminated by an individualized prognostic signature for 5-fluorouracil-based chemotherapy**

Mengsha Tong, Weicheng Zheng, Hongdong Li, Xiangyu Li, Lu Ao, Yifeng Shen, Qirui Liang, Jing Li, Guini Hong, Haidan Yan, Hao Cai, Mengyao Li, Qingzhou Guan, Zheng Guo

**Supplementary Information**

**Supplementary Table S1.** 58 NCI-60 cell lines derived from nine different tumor types.(DOCX)

**Supplementary Table S2.** The enriched pathways of 2588 DEGs between the prognostic groups, consistently extracted from the GSE39582, GSE14333 and TCGA datasets (Wilcoxon rank-sum test, FDR < 10%). P-value was adjusted by Benjamini and Hochberg(FDR<5%).(DOCX )

**Supplementary** **Table S3.** 54 chromosome regions that were significantly higher in the high-risk group than the low-risk group (Fisher-test, FDR<5%).(DOCX )

**Supplementary Table S4.** Genes had copy number alteration or DNA methylation aberration with concordant transcriptional changes in the high-risk group compared with the low-risk group. (DOCX )

**Supplementary Table S5.** The 320 mutation genes exhibited significant higher frequencies in the low-risk group compared with the high-risk group (Fisher-test, P-value<0.05). (DOCX )

**Supplementary Table S6** Genes with mutations involving cell adhesion in the low-risk group. (DOCX)

**Supplementary Table S7.** 1555 DEGs between the prognostic groups(Wilcoxon rank-sum test, FDR<5%) whose expression levels were negatively correlated with their methylation levels (Spearman correlation, FDR<5%).P-value was adjusted by Benjamini and Hochberg (FDR<5%). (DOCX )

**Supplementary Table S8.** 82 genes involved in 5-FU transport, metabolism and other downstream effects(such as DNA repair, apoptosis and cell cycle regulation),denoted as 5-FU activity-related genes, which were collected from a previous study[^1^](#_ENREF_1) (DOCX )

**Supplementary Table S9.** The frequencies of the CMS subtypes predicted by the SSP method in the high-risk/low-risk groups, respectively.

**Supplementary Table S10.** The comparisons between the study reported by Guinney et al and our work.

**Supplementary** **Data1.** The prevalence of copy number alterations for the 12 pathways associated with 5-FU sensitivity among the 91 high-risk samples. (DOCX )

**Supplementary Data2.** The prevalence of mutations for the 12 pathways associated with 5-FU sensitivity among the 68 low-risk samples(XLSX )

**Supplementary Tables**

**Supplementary Table S1**: 58 NCI-60 cell lines derived from nine different tumor types.

| Experiment label | Cell line | -LOG_10_(GI_50_)(μmol/L) |
| --- | --- | --- |
| 13401HG133A21 | LE:CCRF_CEM | 5.01 |
| 13402HG133A21 | LE:HL_60 | 5.64 |
| 13403HG133A21 | LE:MOLT_4 | 6.45 |
| 13404HG133A21 | LE:RPMI_8226 | 7.35 |
| 13405HG133A21 | LE:SR | 7.62 |
| 13406HG133A21 | LE:K_562 | 5.45 |
| 13407HG133A31 | BR:MCF7 | 7.10 |
| 13408HG133A21 | OV:NCI_ADR_RES | 6.50 |
| 13409HG133A21 | BR:MDA_MB_231 | 5.18 |
| 13410HG133A21 | BR:HS578T | 5.01 |
| 13411HG133A21 | ME:MDA_MB_435 | 7.14 |
| 13412HG133A21 | BR:T47D | 5.09 |
| 13413HG133A21 | ME:MDA_N | Not available |
| 13414HG133A21 | BR:BT_549 | 4.97 |
| 13415HG133A21 | CNS:SF_268 | 5.79 |
| 13416HG133A21 | CNS:SF_295 | 6.64 |
| 13417HG133A21 | CNS:SF_539 | 7.20 |
| 13418HG133A21 | CNS:SNB_19 | 5.42 |
| 13419HG133A21 | CNS:SNB_75 | 4.10 |
| 13420HG133A21 | CNS:U251 | 6.04 |
| 13421HG133A21 | CO:COLO205 | 6.80 |
| 13422HG133A21 | CO:HCC_2998 | 7.28 |
| 13423HG133A21 | CO:HCT_116 | 6.64 |
| 13424HG133A21 | CO:HCT_15 | 6.96 |
| 13425HG133A21 | CO:HT29 | 6.75 |
| 13426HG133A21 | CO:KM12 | 6.67 |
| 13427HG133A21 | CO:SW_620 | 6.03 |
| 13428HG133A21 | RE:786_0 | 6.14 |
| 13429HG133A21 | RE:A498 | 6.40 |
| 13430HG133A21 | RE:ACHN | 6.53 |
| 13431HG133A21 | RE:CAKI_1 | 7.14 |
| 13432HG133A21 | RE:RXF_393 | 5.58 |
| 13433HG133A21 | RE:SN12C | 6.30 |
| 13434HG133A21 | RE:TK_10 | 5.95 |
| 13435HG133A21 | RE:UO_31 | 5.85 |
| 13436HG133A21 | LC:A549 | 6.72 |
| 13437HG133A21 | LC:EKVX | 4.21 |
| 13438HG133A21 | LC:HOP_62 | 6.40 |
| 13439HG133A21 | LC:HOP_92 | 4.11 |
| 13440HG133A21 | LC:NCI_H226 | 4.26 |
| Not available | LC:NCI_H23 | Not available |
| 13442HG133A21 | LC:NCI_H322M | 6.75 |
| 13443HG133A21 | LC:NCI_H460 | 7.25 |
| 13444HG133A21 | LC:NCI_H522 | 5.14 |
| 13445HG133A31 | OV:IGROV1 | 5.91 |
| 13446HG133A21 | OV:OVCAR_3 | 7.80 |
| 13447HG133A21 | OV:OVCAR_4 | 5.35 |
| 13448HG133A21 | OV:OVCAR_5 | 4.96 |
| 13449HG133A21 | OV:OVCAR_8 | 5.76 |
| 13450HG133A21 | OV:SK_OV_3 | 4.66 |
| 13451HG133A21 | PR:PC_3 | 5.63 |
| 13452HG133A21 | PR:DU_145 | 6.44 |
| 13453HG133A21 | ME:LOXIMVI | 6.61 |
| 13454HG133A21 | ME:MALME_3M | 7.29 |
| 13455HG133A21 | ME:M14 | 6.01 |
| 13456HG133A21 | ME:SK_MEL_2 | 4.25 |
| 13457HG133A21 | ME:SK_MEL_28 | 5.99 |
| 13458HG133A21 | ME:SK_MEL_5 | 6.33 |
| 13459HG133A21 | ME:UACC_257 | 5.45 |
| 13460HG133A21 | ME:UACC_62 | 6.28 |

Note: LE: Leukemia; BR: Breast; OV Ovarian; ME: Melanoma; CNS: Central nervous system;

CO: Colon; RE: Renal; LC: Non-Small Cell Lung; PR: Prostate.

**Supplementary Table S2**: The enriched pathways of 2588 DEGs between the prognostic groups, consistently extracted from the GSE39582, GSE14333 and TCGA datasets (Wilcoxon rank-sum test,FDR < 10%). *P*-value was adjusted by Benjamini and Hochberg(FDR<5%).

| KEGG Pathway | P-value | Reference |
| --- | --- | --- |
| Ribosome biogenesis in eukaryotes | <1.11E-16 |  |
| Spliceosome | <1.11E-16 |  |
| Glycosaminoglycan biosynthesis - chondroitin sulfate / dermatan sulfate | 7.20E-05 |  |
| Aminoacyl-tRNA biosynthesis | 3.71E-06 |  |
| RNA transport | 5.10E-14 |  |
| **DNA replication** | **1.83E-12** | [^2-4^](#_ENREF_2) |
| **Cell cycle** | 1.07E-11 | [^3^](#_ENREF_3) |
| **Focal adhesion** | 2.04E-10 | [^5^](#_ENREF_5) |
| **ECM-receptor interaction** | **7.21E-10** | [^6^](#_ENREF_6) |
| **Pyrimidine metabolism** | **8.86E-10** | [^7^](#_ENREF_7)^,^ [^8^](#_ENREF_8) |
| Fanconi anemia pathway | 2.01E-07 |  |
| **Base excision repair** | **2.48E-07** | [^9^](#_ENREF_9) |
| Complement and coagulation cascades | 4.81E-07 |  |
| Purine metabolism | 1.30E-06 |  |
| **Mismatch repair** | **3.24E-06** | [^9^](#_ENREF_9)^,^ [^10^](#_ENREF_10) |
| Ribosome | 3.32E-06 |  |
| Phagosome | 7.05E-06 |  |
| **PI3K-Akt signaling pathway** | **1.10E-05** | [^11^](#_ENREF_11)^,^ [^12^](#_ENREF_12) |
| **Cell adhesion molecules (CAMs)** | **2.79E-05** | [^13^](#_ENREF_13)^,^ [^14^](#_ENREF_14) |
| cGMP-PKG signaling pathway | 1.79E-04 |  |
| Biosynthesis of amino acids | 1.91E-04 |  |
| Regulation of actin cytoskeleton | 2.24E-04 |  |
| Proteasome | 2.67E-04 |  |
| Vascular smooth muscle contraction | 3.62E-04 |  |
| Protein digestion and absorption | 4.37E-04 |  |
| **Ras signaling pathway** | **4.48E-04** | [^15^](#_ENREF_15)^,^ [^16^](#_ENREF_16) |
| RNA polymerase | 5.76E-04 |  |
| Carbon metabolism | 5.91E-04 |  |
| Platelet activation | 6.81E-04 |  |
| **Nucleotide excision repair** | **1.02E-03** | [^9^](#_ENREF_9) |
| **Rap1 signaling pathway** | **1.08E-03** | [^17^](#_ENREF_17) |
| Osteoclast differentiation | 1.69E-03 |  |
| Leukocyte transendothelial migration | 1.80E-03 |  |
| mRNA surveillance pathway | 1.97E-03 |  |
| Steroid biosynthesis | 2.04E-03 |  |
| Fructose and mannose metabolism | 4.06E-03 |  |

Note: Bold parts are pathways have been reported to be associated with 5-FU sensitivity.

**Supplementary Table S3**: 54 chromosome regions that were significantly higher in the high-risk group than the low-risk group (Fisher-test, FDR<5%).

|  | Region | P-value | High- risk ^a^ | | High-risk frequency ^b^ | Low- risk ^a^ | Low-risk frequency ^b^ |
| --- | --- | --- | --- | --- | --- | --- | --- |
| Amplification region | 5p13.2 | 3.56E-02 | | 22 | 24.18% | 11 | 11.96% |
|  | 6p21.1 | 2.12E-03 | | 28 | 30.77% | 11 | 11.96% |
|  | 7p21.2 | 3.20E-09 | | 72 | 79.12% | 33 | 35.87% |
|  | 8p11.23 | 9.60E-03 | | 43 | 47.25% | 26 | 28.26% |
|  | 8p11.21 | 1.97E-04 | | 54 | 59.34% | 29 | 31.52% |
|  | 8q12.1 | 3.43E-07 | | 67 | 73.63% | 33 | 35.87% |
|  | 8q24.21 | 1.53E-08 | | 73 | 80.22% | 36 | 39.13% |
|  | 12p13.33 | 9.06E-03 | | 29 | 31.87% | 14 | 15.22% |
|  | 13q12.13 | 1.44E-06 | | 68 | 74.73% | 36 | 39.13% |
|  | 13q22.1 | 5.63E-07 | | 70 | 76.92% | 37 | 40.22% |
|  | 16p11.2 | 6.28E-04 | | 33 | 36.26% | 13 | 14.13% |
|  | 16q12.1 | 1.76E-04 | | 31 | 34.07% | 10 | 10.87% |
|  | 19p13.2 | 6.51E-03 | | 23 | 25.27% | 9 | 9.78% |
|  | 19q13.11 | 7.03E-05 | | 31 | 34.07% | 9 | 9.78% |
|  | 20p12.1 | 1.85E-05 | | 49 | 53.85% | 21 | 22.83% |
|  | 20p11.21 | 1.74E-07 | | 64 | 70.33% | 29 | 31.52% |
|  | 20q11.21 | 7.11E-14 | | 85 | 93.41% | 40 | 43.48% |
|  | 20q12 | 1.34E-15 | | 87 | 95.60% | 40 | 43.48% |
|  | 20q13.12 | 1.34E-15 | | 87 | 95.60% | 40 | 43.48% |
| Deletion region | 1p36.11 | 9.76E-04 | | 41 | 45.05% | 20 | 21.74% |
|  | 1p33 | 7.52E-04 | | 34 | 37.36% | 14 | 15.22% |
|  | 1p13.1 | 1.74E-02 | | 30 | 32.97% | 16 | 17.39% |
|  | 2p21 | 1.39E-02 | | 6 | 6.59% | 0 | 0.00% |
|  | 3p26.2 | 5.14E-03 | | 19 | 20.88% | 6 | 6.52% |
|  | 3p14.2 | 1.27E-02 | | 23 | 25.27% | 10 | 10.87% |
|  | 4p16.2 | 1.27E-04 | | 33 | 36.26% | 11 | 11.96% |
|  | 4q22.1 | 4.08E-05 | | 39 | 42.86% | 14 | 15.22% |
|  | 4q35.1 | 1.47E-03 | | 39 | 42.86% | 19 | 20.65% |
|  | 5q12.1 | 2.90E-03 | | 26 | 28.57% | 10 | 10.87% |
|  | 5q21.1 | 6.28E-04 | | 33 | 36.26% | 13 | 14.13% |
|  | 5q22.2 | 2.47E-04 | | 36 | 39.56% | 14 | 15.22% |
|  | 6p25.3 | 3.89E-02 | | 15 | 16.48% | 6 | 6.52% |
|  | 6q26 | 3.89E-02 | | 15 | 16.48% | 6 | 6.52% |
|  | 8p23.3 | 3.71E-10 | | 58 | 63.74% | 17 | 18.48% |
|  | 8p22 | 2.25E-10 | | 62 | 68.13% | 20 | 21.74% |
|  | 9p21.3 | 1.14E-03 | | 20 | 21.98% | 5 | 5.43% |
|  | 10p15.3 | 6.81E-03 | | 20 | 21.98% | 7 | 7.61% |
|  | 10q21.1 | 3.54E-03 | | 27 | 29.67% | 11 | 11.96% |
|  | 10q23.31 | 8.92E-04 | | 31 | 34.07% | 12 | 13.04% |
|  | 10q25.2 | 1.25E-03 | | 29 | 31.87% | 11 | 11.96% |
|  | 14q31.1 | 8.05E-03 | | 33 | 36.26% | 17 | 18.48% |
|  | 15q11.2 | 1.69E-04 | | 43 | 47.25% | 19 | 20.65% |
|  | 15q15.2 | 2.67E-05 | | 41 | 45.05% | 15 | 16.30% |
|  | 15q21.1 | 4.99E-05 | | 40 | 43.96% | 15 | 16.30% |
|  | 15q22.33 | 1.10E-04 | | 40 | 43.96% | 16 | 17.39% |
|  | 16p13.3 | 3.89E-02 | | 15 | 16.48% | 6 | 6.52% |
|  | 17p12 | 1.64E-07 | | 66 | 72.53% | 31 | 33.70% |
|  | 17q24.3 | 8.76E-03 | | 18 | 19.78% | 6 | 6.52% |
|  | 18p11.31 | 7.43E-08 | | 65 | 71.43% | 29 | 31.52% |
|  | 18q12.2 | 9.30E-10 | | 74 | 81.32% | 34 | 36.96% |
|  | 18q21.2 | 3.58E-12 | | 80 | 87.91% | 36 | 39.13% |
|  | 20p12.1 | 7.94E-03 | | 28 | 30.77% | 13 | 14.13% |
|  | 21q11.2 | 2.18E-03 | | 37 | 40.66% | 18 | 19.57% |
|  | 22q13.32 | 4.01E-04 | | 39 | 42.86% | 17 | 18.48% |

^a^The number of samples with CNV in the high-risk/low-risk group ^b^The CNV frequencies in the high-risk/low-risk group

**Supplementary Table S4**: Genes had copy number alteration or DNA methylation aberration with concordant transcriptional changes in the high-risk group compared with the low-risk group.

| Gene symbol | copy number alteration | DNA methylation aberration | Pathway |
| --- | --- | --- | --- |
| DCTD | ↓ |  | Pyrimidine metabolism |
| TWISTNB | ↓ |  | Pyrimidine metabolism |
| NT5C2 | ↓ |  | Pyrimidine metabolism |
| NT5C3A | ↓ |  | Pyrimidine metabolism |
| POLR1C | ↑ |  | Pyrimidine metabolism |
| DPYD | ↑ |  | Pyrimidine metabolism |
| TYMP | ↓ |  | Pyrimidine metabolism |
| TGFB3 | ↓ |  | Cell cycle |
| SMAD3 | ↓ |  | Cell cycle |
| SMAD4 | ↓ |  | Cell cycle |
| SFN | ↓ |  | Cell cycle |
| MYC | ↑ |  | Cell cycle /PI3K-Akt signaling |
| PRKDC | ↑ |  | Cell cycle /PI3K-Akt signaling |
| BUB1B | ↓ |  | Cell cycle |
| GADD45A | ↓ |  | Cell cycle |
| MCM4 | ↑ |  | Cell cycle /DNA replication |
| RBL1 | ↑ |  | Cell cycle |
| SKP2 | ↑ |  | Cell cycle |
| CDC7 | ↓ |  | Cell cycle |
| MAD1L1 | ↑ |  | Cell cycle |
| CDC14A | ↓ |  | Cell cycle |
| CCND2 | ↓ |  | Cell cycle /PI3K-Akt signaling/Focal adhesion |
| CCND3 | ↓ |  | Cell cycle /PI3K-Akt signaling/Focal adhesion |
| SMC3 | ↓ |  | Cell cycle |
| BUB3 | ↓ |  | Cell cycle |
| CHUK | ↓ |  | PI3K-Akt signaling/Ras signaling |
| EFNA5 | ↓ |  | PI3K-Akt signaling/Ras signaling/Rap1 signaling |
| AKT1 | ↓ |  | PI3K-Akt signaling/Ras signaling/Rap1 signaling /Focal adhesion |
| FGF10 | ↑ |  | PI3K-Akt signaling |
| FGFR2 | ↓ |  | PI3K-Akt signaling |
| SGK3 | ↑ |  | PI3K-Akt signaling |
| GNG5 | ↓ |  | PI3K-Akt signaling/Ras signaling |
| HSP90AA1 | ↑ |  | PI3K-Akt signaling |
| HSP90AB1 | ↑ |  | PI3K-Akt signaling |
| IKBKB | ↑ |  | PI3K-Akt signaling/Ras signaling |
| JAK1 | ↓ |  | PI3K-Akt signaling |
| NRAS | ↓ |  | PI3K-Akt signalingRas signaling/Rap1 signaling |
| **PDGFA** | ↑ | ↑ | PI3K-Akt signaling/RAS signaling/ Rap1 signaling /Focal adhesion |
| PPP2R5C | ↓ |  | PI3K-Akt signaling |
| PPP2R5D | ↓ |  | PI3K-Akt signaling |
| PPP2R5E | ↓ |  | PI3K-Akt signaling |
| PPP2R2D | ↓ |  | PI3K-Akt signaling |
| PKN2 | ↓ |  | PI3K-Akt signaling |
| GNG12 | ↓ |  | PI3K-Akt signaling/Ras signaling |
| PRLR | ↑ |  | PI3K-Akt signaling |
| PTEN | ↓ |  | PI3K-Akt signaling/Focal adhesion |
| RAC1 | ↑ |  | PI3K-Akt signaling/RAS signaling/ Rap1 signaling /Focal adhesion |
| RAF1 | ↓ |  | PI3K-Akt signaling/RAS signaling/ Rap1 signaling /Focal adhesion |
| BCL2L1 | ↑ |  | PI3K-Akt signaling/Ras signaling |
| VEGFA | ↑ |  | PI3K-Akt signaling/Ras signaling/Focal adhesion/Rap1 signaling |
| TCL1B | ↓ |  | PI3K-Akt signaling |
| **ANGPT1** | ↑ | ↑ | PI3K-Akt signaling/Rap1 signaling |
| COL4A1 |  | ↑ | PI3K-Akt signaling |
| COL4A3 |  | ↑ | PI3K-Akt signaling |
| COL4A6 |  | ↑ | PI3K-Akt signaling |
| COL5A1 |  | ↑ | PI3K-Akt signaling |
| COL11A1 |  | ↑ | PI3K-Akt signaling |
| **CSF1R** | ↑ | ↑ | PI3K-Akt signaling/Rap1 signaling |
| CSF3R |  | ↑ | PI3K-Akt signaling |
| **F2R** | ↑ | ↑ | PI3K-Akt signaling/Rap1 signaling |
| **FGF1** | ↑ | ↑ | PI3K-Akt signaling/Rap1 signaling |
| FGF2 |  | ↑ | PI3K-Akt signaling |
| **FGF5** | ↑ | ↑ | PI3K-Akt signaling/Rap1 signaling |
| FGF10 5p13.2 |  | ↑ | PI3K-Akt signaling/Ras signaling/Rap1 signaling |
| **FGF12** | ↑ | ↑ | PI3K-Akt signaling/Rap1 signaling |
| **FGFR1** | ↑ | ↑ | PI3K-Akt signaling/Rap1 signaling |
| **FLT4** | ↑ | ↑ | PI3K-Akt signaling/Rap1 signaling |
| FN1 |  | ↑ | PI3K-Akt signaling |
| **IGF1** | ↑ | ↑ | PI3K-Akt signaling/Rap1 signaling |
| IL2RB |  | ↑ | PI3K-Akt signaling |
| IL6R |  | ↑ | PI3K-Akt signaling |
| **ITGA4** | ↑ | ↑ | PI3K-Akt signaling/Cell adhesion molecules (CAMs) |
| **ITGA9** | ↑ | ↑ | PI3K-Akt signaling/Cell adhesion molecules (CAMs) |
| **ITGB8** | ↑ | ↑ | PI3K-Akt signaling/Cell adhesion molecules (CAMs) |
| **EFNA2** | ↓ | ↓ | PI3K-Akt signaling/Rap1 signaling |
| PPP2R2A |  | ↓ | PI3K-Akt signaling |
| **PRKCZ** | ↓ | ↓ | PI3K-Akt signaling/Rap1 signaling |
| **KDR** | ↑ | ↑ | PI3K-Akt signaling/Rap1 signaling |
| NGF |  | ↑ | PI3K-Akt signaling/Rap1 signaling |
| **PDGFRB** | ↑ | ↑ | PI3K-Akt signaling/Rap1 signaling |
| **PIK3CD** |  | ↑ | PI3K-Akt signaling/Rap1 signaling |
| **PIK3CG** | ↑ | ↑ | PI3K-Akt signaling/Rap1 signaling |
| PPP2R5C 14q31.1 |  | ↑ | PI3K-Akt signaling |
| FGFR2 | ↓ |  | Ras signaling |
| PLA2G4D | ↓ |  | Ras signaling |
| PLCE1 | ↓ |  | Ras signaling/Rap1 signaling |
| PLCG1 | ↑ |  | Ras signaling/Rap1 signaling |
| PAK6 | ↓ |  | Ras signaling/Focal adhesion |
| RAB5A | ↓ |  | Ras signaling |
| RALA | ↑ |  | Ras signaling/Rap1 signaling pathway |
| RAP1A | ↓ |  | Ras signaling/Focal adhesion /Rap1 signaling |
| CALM1 | ↓ |  | Ras signaling/Rap1 signaling |
| RAPGEF5 | ↑ |  | Ras signaling/Rap1 signaling |
| ACTB | ↑ |  | Rap1 signaling /Focal adhesion |
| ACTN1 | ↓ |  | Focal adhesion |
| SRC | ↑ |  | Focal adhesion/Rap1 signaling |
| GNAI3 | ↓ |  | Rap1 signaling |
| ID1 | ↑ |  | Rap1 signaling |
| MAPK11 | ↓ |  | Rap1 signaling |
| MAPK12 | ↓ |  | Rap1 signaling |
| SIPA1L1 | ↓ |  | Rap1 signaling |
| PRKD1 | ↓ |  | Rap1 signaling |
| SIPA1 | ↓ |  | Rap1 signaling |
| TEK | ↓ |  | Rap1 signaling |
| TIAM1 | ↓ |  | Rap1 signaling |
| VEGFB | ↓ |  | Rap1 signaling |
| VEGFC | ↓ |  | Rap1 signaling |
| RASGRP2 | ↓ |  | Rap1 signaling |
| RAPGEF3 | ↓ |  | Rap1 signaling |
| LAT | ↓ |  | Rap1 signaling |
| ARAP3 | ↓ |  | Rap1 signaling |
| PDGFD | ↓ |  | Rap1 signaling |
| ARAP1 | ↓ |  | Rap1 signaling |
| RPA3 | ↑ |  | DNA replication/Mismatch repair/Nucleotide excision repair |
| MLH3 | ↓ |  | Mismatch repair |
| PMS2 | ↑ |  | Mismatch repair |
| MNAT1 | ↓ |  | Nucleotide excision repair |
| XPC | ↓ |  | Nucleotide excision repair |
| POLB | ↑ |  | Base excision repair |
| POLL | ↓ |  | Base excision repair |
| CD58 |  | ↑ | Cell adhesion molecules (CAMs) |
| CD6 |  | ↑ | Cell adhesion molecules (CAMs) |
| CD28 |  | ↑ | Cell adhesion molecules (CAMs) |
| CD34 |  | ↑ | Cell adhesion molecules (CAMs) |
| CDH2 |  | ↑ | Cell adhesion molecules (CAMs) |
| HLA-DOB |  | ↑ | Cell adhesion molecules (CAMs) |
| HLA-DPB1 |  | ↑ | Cell adhesion molecules (CAMs) |
| ICAM2 |  | ↑ | Cell adhesion molecules (CAMs) |
| ICAM3 |  | ↑ | Cell adhesion molecules (CAMs) |
| NCAM1 |  | ↑ | Cell adhesion molecules (CAMs) |
| NCAM2 |  | ↑ | Cell adhesion molecules (CAMs) |
| PTPRC |  | ↑ | Cell adhesion molecules (CAMs) |
| PTPRM |  | ↑ | Cell adhesion molecules (CAMs) |
| SELE |  | ↑ | Cell adhesion molecules (CAMs) |
| SELPLG |  | ↑ | Cell adhesion molecules (CAMs) |
| SPN |  | ↑ | Cell adhesion molecules (CAMs) |
| MPZL1 |  | ↑ | Cell adhesion molecules (CAMs) |
| CD226 |  | ↑ | Cell adhesion molecules (CAMs) |
| NTNG1 |  | ↑ | Cell adhesion molecules (CAMs) |
| NFASC |  | ↑ | Cell adhesion molecules (CAMs) |
| ICOS |  | ↑ | Cell adhesion molecules (CAMs) |
| NLGN4X |  | ↑ | Cell adhesion molecules (CAMs) |
| JAM2 |  | ↑ | Cell adhesion molecules (CAMs) |
| LRRC4 |  | ↑ | Cell adhesion molecules (CAMs) |
| JAM3 |  | ↑ | Cell adhesion molecules (CAMs) |
| TIGIT |  | ↑ | Cell adhesion molecules (CAMs) |
| NEGR1 |  | ↑ | Cell adhesion molecules (CAMs) |
| CLDN3 |  | ↓ | Cell adhesion molecules (CAMs) |

Note: Bold parts are genes both with copy number alterations and DNA methylation aberrations.

↑:amplification/hypomethylation- up-regulated gene; ↓:deletion/hypermethylation-down-regulate gene.

**Supplementary Table S5**: The 320 mutation genes exhibited significant higher frequencies in the low-risk group compared with the high-risk group (Fisher-test, *P*-value<0.05).

| Entrez gene ID | P-value | High- risk ^a^ | High-risk frequency ^b^ | Low- risk ^a^ | Low-risk frequency ^b^ |
| --- | --- | --- | --- | --- | --- |
| 10060 | 1.02E-02 | 0 | 0.00% | 6 | 8.82% |
| 101 | 1.02E-02 | 0 | 0.00% | 6 | 8.82% |
| 10117 | 2.56E-02 | 2 | 2.67% | 9 | 13.24% |
| 10142 | 2.44E-02 | 5 | 6.67% | 14 | 20.59% |
| 10188 | 4.88E-02 | 0 | 0.00% | 4 | 5.88% |
| 1025 | 4.88E-02 | 0 | 0.00% | 4 | 5.88% |
| 10256 | 4.88E-02 | 0 | 0.00% | 4 | 5.88% |
| 1045 | 4.88E-02 | 0 | 0.00% | 4 | 5.88% |
| 10500 | 4.88E-02 | 0 | 0.00% | 4 | 5.88% |
| 10505 | 4.88E-02 | 0 | 0.00% | 4 | 5.88% |
| 10794 | 4.88E-02 | 0 | 0.00% | 4 | 5.88% |
| 10897 | 4.64E-03 | 0 | 0.00% | 7 | 10.29% |
| 10908 | 2.25E-02 | 0 | 0.00% | 5 | 7.35% |
| 11000 | 2.25E-02 | 0 | 0.00% | 5 | 7.35% |
| 11086 | 4.64E-03 | 0 | 0.00% | 7 | 10.29% |
| 11095 | 4.88E-02 | 0 | 0.00% | 4 | 5.88% |
| 1113 | 4.64E-03 | 0 | 0.00% | 7 | 10.29% |
| 11176 | 4.88E-02 | 0 | 0.00% | 4 | 5.88% |
| 112476 | 2.25E-02 | 0 | 0.00% | 5 | 7.35% |
| 1131 | 4.88E-02 | 0 | 0.00% | 4 | 5.88% |
| 11335 | 4.88E-02 | 0 | 0.00% | 4 | 5.88% |
| 113730 | 1.02E-02 | 0 | 0.00% | 6 | 8.82% |
| 114785 | 2.56E-02 | 2 | 2.67% | 9 | 13.24% |
| 115752 | 6.74E-03 | 1 | 1.33% | 9 | 13.24% |
| 117583 | 4.88E-02 | 0 | 0.00% | 4 | 5.88% |
| 120071 | 4.88E-02 | 0 | 0.00% | 4 | 5.88% |
| 124245 | 4.74E-02 | 2 | 2.67% | 8 | 11.76% |
| 125950 | 4.88E-02 | 0 | 0.00% | 4 | 5.88% |
| 126669 | 4.88E-02 | 0 | 0.00% | 4 | 5.88% |
| 127534 | 4.88E-02 | 0 | 0.00% | 4 | 5.88% |
| 1294 | 2.44E-02 | 5 | 6.67% | 14 | 20.59% |
| 131377 | 4.88E-02 | 0 | 0.00% | 4 | 5.88% |
| 140775 | 4.88E-02 | 0 | 0.00% | 4 | 5.88% |
| 146 | 4.88E-02 | 0 | 0.00% | 4 | 5.88% |
| 146212 | 1.02E-02 | 0 | 0.00% | 6 | 8.82% |
| 146540 | 2.25E-02 | 0 | 0.00% | 5 | 7.35% |
| 149076 | 4.88E-02 | 0 | 0.00% | 4 | 5.88% |
| 149628 | 4.88E-02 | 0 | 0.00% | 4 | 5.88% |
| 150483 | 1.02E-02 | 0 | 0.00% | 6 | 8.82% |
| 150946 | 2.25E-02 | 0 | 0.00% | 5 | 7.35% |
| 152503 | 4.88E-02 | 0 | 0.00% | 4 | 5.88% |
| 153090 | 1.02E-02 | 0 | 0.00% | 6 | 8.82% |
| 155054 | 4.88E-02 | 0 | 0.00% | 4 | 5.88% |
| 155061 | 2.25E-02 | 0 | 0.00% | 5 | 7.35% |
| 157983 | 2.25E-02 | 0 | 0.00% | 5 | 7.35% |
| 164832 | 4.88E-02 | 0 | 0.00% | 4 | 5.88% |
| 1674 | 4.88E-02 | 0 | 0.00% | 4 | 5.88% |
| 168090 | 2.73E-02 | 1 | 1.33% | 7 | 10.29% |
| 168667 | 2.25E-02 | 0 | 0.00% | 5 | 7.35% |
| 169044 | 1.56E-03 | 1 | 1.33% | 11 | 16.18% |
| 1808 | 4.88E-02 | 0 | 0.00% | 4 | 5.88% |
| 1820 | 4.88E-02 | 0 | 0.00% | 4 | 5.88% |
| 1822 | 3.27E-03 | 1 | 1.33% | 10 | 14.71% |
| 1879 | 4.88E-02 | 0 | 0.00% | 4 | 5.88% |
| 1936 | 2.08E-03 | 0 | 0.00% | 8 | 11.76% |
| 1951 | 2.08E-03 | 0 | 0.00% | 8 | 11.76% |
| 1953 | 4.74E-02 | 2 | 2.67% | 8 | 11.76% |
| 200150 | 4.88E-02 | 0 | 0.00% | 4 | 5.88% |
| 2035 | 4.88E-02 | 0 | 0.00% | 4 | 5.88% |
| 205860 | 4.88E-02 | 0 | 0.00% | 4 | 5.88% |
| 2101 | 4.88E-02 | 0 | 0.00% | 4 | 5.88% |
| 2194 | 1.79E-02 | 4 | 5.33% | 13 | 19.12% |
| 2195 | 1.79E-02 | 4 | 5.33% | 13 | 19.12% |
| 221477 | 4.88E-02 | 0 | 0.00% | 4 | 5.88% |
| 221785 | 4.88E-02 | 0 | 0.00% | 4 | 5.88% |
| 2258 | 4.88E-02 | 0 | 0.00% | 4 | 5.88% |
| 22853 | 4.88E-02 | 0 | 0.00% | 4 | 5.88% |
| 22905 | 4.88E-02 | 0 | 0.00% | 4 | 5.88% |
| 2295 | 4.88E-02 | 0 | 0.00% | 4 | 5.88% |
| 22982 | 2.56E-02 | 2 | 2.67% | 9 | 13.24% |
| 22994 | 2.73E-02 | 1 | 1.33% | 7 | 10.29% |
| 23094 | 4.74E-02 | 2 | 2.67% | 8 | 11.76% |
| 23096 | 2.73E-02 | 1 | 1.33% | 7 | 10.29% |
| 23116 | 2.08E-03 | 0 | 0.00% | 8 | 11.76% |
| 23122 | 4.88E-02 | 0 | 0.00% | 4 | 5.88% |
| 23130 | 1.37E-02 | 1 | 1.33% | 8 | 11.76% |
| 23158 | 4.64E-03 | 0 | 0.00% | 7 | 10.29% |
| 2316 | 3.27E-03 | 1 | 1.33% | 10 | 14.71% |
| 23166 | 3.92E-02 | 3 | 4.00% | 10 | 14.71% |
| 2317 | 2.56E-02 | 2 | 2.67% | 9 | 13.24% |
| 23185 | 4.74E-02 | 2 | 2.67% | 8 | 11.76% |
| 23194 | 4.88E-02 | 0 | 0.00% | 4 | 5.88% |
| 23198 | 3.27E-03 | 1 | 1.33% | 10 | 14.71% |
| 23232 | 4.88E-02 | 0 | 0.00% | 4 | 5.88% |
| 23245 | 2.73E-02 | 1 | 1.33% | 7 | 10.29% |
| 23251 | 2.56E-02 | 2 | 2.67% | 9 | 13.24% |
| 23258 | 4.88E-02 | 0 | 0.00% | 4 | 5.88% |
| 23261 | 4.74E-02 | 2 | 2.67% | 8 | 11.76% |
| 23373 | 9.25E-04 | 0 | 0.00% | 9 | 13.24% |
| 23396 | 1.02E-02 | 0 | 0.00% | 6 | 8.82% |
| 23559 | 2.25E-02 | 0 | 0.00% | 5 | 7.35% |
| 23678 | 4.88E-02 | 0 | 0.00% | 4 | 5.88% |
| 25 | 4.88E-02 | 0 | 0.00% | 4 | 5.88% |
| 253461 | 4.88E-02 | 0 | 0.00% | 4 | 5.88% |
| 254428 | 2.25E-02 | 0 | 0.00% | 5 | 7.35% |
| 255057 | 2.25E-02 | 0 | 0.00% | 5 | 7.35% |
| 257101 | 4.88E-02 | 0 | 0.00% | 4 | 5.88% |
| 257240 | 1.37E-02 | 1 | 1.33% | 8 | 11.76% |
| 257364 | 4.88E-02 | 0 | 0.00% | 4 | 5.88% |
| 2583 | 2.08E-03 | 0 | 0.00% | 8 | 11.76% |
| 25878 | 1.37E-02 | 1 | 1.33% | 8 | 11.76% |
| 25921 | 4.88E-02 | 0 | 0.00% | 4 | 5.88% |
| 25959 | 4.88E-02 | 0 | 0.00% | 4 | 5.88% |
| 25963 | 4.88E-02 | 0 | 0.00% | 4 | 5.88% |
| 26013 | 4.88E-02 | 0 | 0.00% | 4 | 5.88% |
| 26051 | 2.25E-02 | 0 | 0.00% | 5 | 7.35% |
| 26058 | 3.27E-03 | 1 | 1.33% | 10 | 14.71% |
| 26153 | 4.74E-02 | 2 | 2.67% | 8 | 11.76% |
| 26173 | 1.02E-02 | 0 | 0.00% | 6 | 8.82% |
| 26240 | 4.88E-02 | 0 | 0.00% | 4 | 5.88% |
| 2625 | 4.88E-02 | 0 | 0.00% | 4 | 5.88% |
| 26470 | 4.88E-02 | 0 | 0.00% | 4 | 5.88% |
| 2650 | 4.88E-02 | 0 | 0.00% | 4 | 5.88% |
| 26996 | 4.88E-02 | 0 | 0.00% | 4 | 5.88% |
| 27043 | 1.37E-02 | 1 | 1.33% | 8 | 11.76% |
| 27132 | 4.88E-02 | 0 | 0.00% | 4 | 5.88% |
| 27134 | 2.25E-02 | 0 | 0.00% | 5 | 7.35% |
| 27153 | 2.25E-02 | 0 | 0.00% | 5 | 7.35% |
| 27239 | 2.25E-02 | 0 | 0.00% | 5 | 7.35% |
| 27252 | 4.88E-02 | 0 | 0.00% | 4 | 5.88% |
| 27332 | 4.88E-02 | 0 | 0.00% | 4 | 5.88% |
| 2802 | 4.88E-02 | 0 | 0.00% | 4 | 5.88% |
| 283234 | 4.74E-02 | 2 | 2.67% | 8 | 11.76% |
| 283373 | 4.88E-02 | 0 | 0.00% | 4 | 5.88% |
| 284086 | 1.02E-02 | 0 | 0.00% | 6 | 8.82% |
| 2849 | 4.88E-02 | 0 | 0.00% | 4 | 5.88% |
| 285440 | 4.88E-02 | 0 | 0.00% | 4 | 5.88% |
| 286319 | 2.25E-02 | 0 | 0.00% | 5 | 7.35% |
| 28992 | 4.88E-02 | 0 | 0.00% | 4 | 5.88% |
| 2915 | 2.56E-02 | 2 | 2.67% | 9 | 13.24% |
| 2923 | 4.88E-02 | 0 | 0.00% | 4 | 5.88% |
| 29926 | 4.88E-02 | 0 | 0.00% | 4 | 5.88% |
| 29998 | 2.08E-03 | 0 | 0.00% | 8 | 11.76% |
| 3081 | 4.88E-02 | 0 | 0.00% | 4 | 5.88% |
| 30811 | 2.25E-02 | 0 | 0.00% | 5 | 7.35% |
| 3106 | 1.36E-02 | 2 | 2.67% | 10 | 14.71% |
| 3146 | 4.88E-02 | 0 | 0.00% | 4 | 5.88% |
| 3212 | 4.88E-02 | 0 | 0.00% | 4 | 5.88% |
| 3321 | 2.56E-02 | 2 | 2.67% | 9 | 13.24% |
| 3339 | 1.20E-02 | 3 | 4.00% | 12 | 17.65% |
| 338321 | 1.37E-02 | 1 | 1.33% | 8 | 11.76% |
| 339896 | 4.88E-02 | 0 | 0.00% | 4 | 5.88% |
| 3416 | 4.88E-02 | 0 | 0.00% | 4 | 5.88% |
| 3430 | 4.88E-02 | 0 | 0.00% | 4 | 5.88% |
| 3549 | 4.88E-02 | 0 | 0.00% | 4 | 5.88% |
| 357 | 2.25E-02 | 0 | 0.00% | 5 | 7.35% |
| 3675 | 4.88E-02 | 0 | 0.00% | 4 | 5.88% |
| 3691 | 4.74E-02 | 2 | 2.67% | 8 | 11.76% |
| 3728 | 4.88E-02 | 0 | 0.00% | 4 | 5.88% |
| 374 | 1.42E-02 | 5 | 6.67% | 15 | 22.06% |
| 374877 | 4.88E-02 | 0 | 0.00% | 4 | 5.88% |
| 375033 | 1.02E-02 | 0 | 0.00% | 6 | 8.82% |
| 375790 | 4.64E-03 | 0 | 0.00% | 7 | 10.29% |
| 388323 | 1.37E-02 | 1 | 1.33% | 8 | 11.76% |
| 388960 | 4.88E-02 | 0 | 0.00% | 4 | 5.88% |
| 3897 | 4.88E-02 | 0 | 0.00% | 4 | 5.88% |
| 391109 | 4.88E-02 | 0 | 0.00% | 4 | 5.88% |
| 3912 | 2.73E-02 | 1 | 1.33% | 7 | 10.29% |
| 4125 | 4.88E-02 | 0 | 0.00% | 4 | 5.88% |
| 4130 | 1.02E-02 | 0 | 0.00% | 6 | 8.82% |
| 4176 | 4.64E-03 | 0 | 0.00% | 7 | 10.29% |
| 4287 | 2.25E-02 | 0 | 0.00% | 5 | 7.35% |
| 440073 | 2.56E-02 | 2 | 2.67% | 9 | 13.24% |
| 442425 | 2.25E-02 | 0 | 0.00% | 5 | 7.35% |
| 4580 | 4.74E-02 | 2 | 2.67% | 8 | 11.76% |
| 4583 | 4.74E-02 | 2 | 2.67% | 8 | 11.76% |
| 4585 | 3.96E-02 | 7 | 9.33% | 15 | 22.06% |
| 4607 | 4.88E-02 | 0 | 0.00% | 4 | 5.88% |
| 4608 | 4.88E-02 | 0 | 0.00% | 4 | 5.88% |
| 4640 | 2.25E-02 | 0 | 0.00% | 5 | 7.35% |
| 4647 | 2.56E-02 | 2 | 2.67% | 9 | 13.24% |
| 5045 | 4.88E-02 | 0 | 0.00% | 4 | 5.88% |
| 50485 | 2.73E-02 | 1 | 1.33% | 7 | 10.29% |
| 50506 | 2.25E-02 | 0 | 0.00% | 5 | 7.35% |
| 50509 | 2.56E-02 | 2 | 2.67% | 9 | 13.24% |
| 5098 | 4.88E-02 | 0 | 0.00% | 4 | 5.88% |
| 51105 | 2.25E-02 | 0 | 0.00% | 5 | 7.35% |
| 51332 | 2.56E-02 | 2 | 2.67% | 9 | 13.24% |
| 5145 | 2.25E-02 | 0 | 0.00% | 5 | 7.35% |
| 51725 | 2.08E-03 | 0 | 0.00% | 8 | 11.76% |
| 5208 | 4.88E-02 | 0 | 0.00% | 4 | 5.88% |
| 5214 | 2.73E-02 | 1 | 1.33% | 7 | 10.29% |
| 5313 | 4.88E-02 | 0 | 0.00% | 4 | 5.88% |
| 5361 | 2.56E-02 | 2 | 2.67% | 9 | 13.24% |
| 5365 | 3.92E-02 | 3 | 4.00% | 10 | 14.71% |
| 53834 | 4.88E-02 | 0 | 0.00% | 4 | 5.88% |
| 5424 | 2.56E-02 | 2 | 2.67% | 9 | 13.24% |
| 5442 | 1.79E-02 | 4 | 5.33% | 13 | 19.12% |
| 54434 | 4.88E-02 | 0 | 0.00% | 4 | 5.88% |
| 54495 | 4.88E-02 | 0 | 0.00% | 4 | 5.88% |
| 54806 | 4.88E-02 | 0 | 0.00% | 4 | 5.88% |
| 54811 | 4.88E-02 | 0 | 0.00% | 4 | 5.88% |
| 54843 | 4.88E-02 | 0 | 0.00% | 4 | 5.88% |
| 54872 | 4.88E-02 | 0 | 0.00% | 4 | 5.88% |
| 54887 | 4.88E-02 | 0 | 0.00% | 4 | 5.88% |
| 54905 | 4.88E-02 | 0 | 0.00% | 4 | 5.88% |
| 54954 | 1.37E-02 | 1 | 1.33% | 8 | 11.76% |
| 55624 | 4.88E-02 | 0 | 0.00% | 4 | 5.88% |
| 55627 | 4.88E-02 | 0 | 0.00% | 4 | 5.88% |
| 55661 | 4.88E-02 | 0 | 0.00% | 4 | 5.88% |
| 55718 | 2.25E-02 | 0 | 0.00% | 5 | 7.35% |
| 55758 | 4.88E-02 | 0 | 0.00% | 4 | 5.88% |
| 55764 | 4.88E-02 | 0 | 0.00% | 4 | 5.88% |
| 5583 | 4.88E-02 | 0 | 0.00% | 4 | 5.88% |
| 55841 | 2.25E-02 | 0 | 0.00% | 5 | 7.35% |
| 5613 | 4.88E-02 | 0 | 0.00% | 4 | 5.88% |
| 56147 | 2.56E-02 | 2 | 2.67% | 9 | 13.24% |
| 56889 | 4.88E-02 | 0 | 0.00% | 4 | 5.88% |
| 56896 | 4.88E-02 | 0 | 0.00% | 4 | 5.88% |
| 56970 | 4.88E-02 | 0 | 0.00% | 4 | 5.88% |
| 56986 | 4.88E-02 | 0 | 0.00% | 4 | 5.88% |
| 57017 | 4.88E-02 | 0 | 0.00% | 4 | 5.88% |
| 57139 | 2.91E-02 | 6 | 8.00% | 0 | 0.00% |
| 57158 | 4.88E-02 | 0 | 0.00% | 4 | 5.88% |
| 57338 | 2.25E-02 | 0 | 0.00% | 5 | 7.35% |
| 57472 | 4.88E-02 | 0 | 0.00% | 4 | 5.88% |
| 57479 | 4.74E-02 | 2 | 2.67% | 8 | 11.76% |
| 57480 | 4.88E-02 | 0 | 0.00% | 4 | 5.88% |
| 57495 | 2.73E-02 | 1 | 1.33% | 7 | 10.29% |
| 57512 | 4.74E-02 | 2 | 2.67% | 8 | 11.76% |
| 57576 | 2.25E-02 | 0 | 0.00% | 5 | 7.35% |
| 57631 | 2.73E-02 | 1 | 1.33% | 7 | 10.29% |
| 57634 | 2.19E-02 | 3 | 4.00% | 11 | 16.18% |
| 57661 | 4.88E-02 | 0 | 0.00% | 4 | 5.88% |
| 57666 | 4.88E-02 | 0 | 0.00% | 4 | 5.88% |
| 57704 | 1.02E-02 | 0 | 0.00% | 6 | 8.82% |
| 5802 | 6.74E-03 | 1 | 1.33% | 9 | 13.24% |
| 5834 | 4.74E-02 | 2 | 2.67% | 8 | 11.76% |
| 593 | 4.88E-02 | 0 | 0.00% | 4 | 5.88% |
| 5993 | 2.25E-02 | 0 | 0.00% | 5 | 7.35% |
| 6007 | 4.88E-02 | 0 | 0.00% | 4 | 5.88% |
| 60385 | 2.73E-02 | 1 | 1.33% | 7 | 10.29% |
| 6059 | 4.88E-02 | 0 | 0.00% | 4 | 5.88% |
| 6305 | 6.74E-03 | 1 | 1.33% | 9 | 13.24% |
| 6337 | 2.25E-02 | 0 | 0.00% | 5 | 7.35% |
| 6339 | 2.25E-02 | 0 | 0.00% | 5 | 7.35% |
| 6397 | 2.25E-02 | 0 | 0.00% | 5 | 7.35% |
| 64410 | 4.88E-02 | 0 | 0.00% | 4 | 5.88% |
| 64428 | 4.88E-02 | 0 | 0.00% | 4 | 5.88% |
| 64599 | 2.56E-02 | 2 | 2.67% | 9 | 13.24% |
| 64651 | 4.88E-02 | 0 | 0.00% | 4 | 5.88% |
| 64857 | 4.74E-02 | 2 | 2.67% | 8 | 11.76% |
| 6526 | 4.88E-02 | 0 | 0.00% | 4 | 5.88% |
| 6533 | 4.88E-02 | 0 | 0.00% | 4 | 5.88% |
| 6543 | 2.73E-02 | 1 | 1.33% | 7 | 10.29% |
| 6559 | 4.88E-02 | 0 | 0.00% | 4 | 5.88% |
| 659 | 2.19E-02 | 3 | 4.00% | 11 | 16.18% |
| 6927 | 2.25E-02 | 0 | 0.00% | 5 | 7.35% |
| 7038 | 3.92E-02 | 3 | 4.00% | 10 | 14.71% |
| 7109 | 2.25E-02 | 0 | 0.00% | 5 | 7.35% |
| 7127 | 4.64E-03 | 0 | 0.00% | 7 | 10.29% |
| 716 | 4.64E-03 | 0 | 0.00% | 7 | 10.29% |
| 7249 | 4.88E-02 | 0 | 0.00% | 4 | 5.88% |
| 727897 | 1.43E-02 | 7 | 9.33% | 17 | 25.00% |
| 728215 | 4.88E-02 | 0 | 0.00% | 4 | 5.88% |
| 728378 | 4.88E-02 | 0 | 0.00% | 4 | 5.88% |
| 730051 | 4.59E-02 | 8 | 10.67% | 16 | 23.53% |
| 7343 | 4.88E-02 | 0 | 0.00% | 4 | 5.88% |
| 7407 | 1.02E-02 | 0 | 0.00% | 6 | 8.82% |
| 7450 | 3.92E-02 | 3 | 4.00% | 10 | 14.71% |
| 7629 | 4.88E-02 | 0 | 0.00% | 4 | 5.88% |
| 7764 | 4.88E-02 | 0 | 0.00% | 4 | 5.88% |
| 7866 | 1.02E-02 | 0 | 0.00% | 6 | 8.82% |
| 79039 | 1.02E-02 | 0 | 0.00% | 6 | 8.82% |
| 79057 | 2.25E-02 | 0 | 0.00% | 5 | 7.35% |
| 79142 | 4.88E-02 | 0 | 0.00% | 4 | 5.88% |
| 79414 | 4.88E-02 | 0 | 0.00% | 4 | 5.88% |
| 79671 | 2.08E-03 | 0 | 0.00% | 8 | 11.76% |
| 79759 | 2.25E-02 | 0 | 0.00% | 5 | 7.35% |
| 79780 | 4.88E-02 | 0 | 0.00% | 4 | 5.88% |
| 79784 | 2.56E-02 | 2 | 2.67% | 9 | 13.24% |
| 79803 | 4.88E-02 | 0 | 0.00% | 4 | 5.88% |
| 79915 | 1.36E-02 | 2 | 2.67% | 10 | 14.71% |
| 79924 | 4.88E-02 | 0 | 0.00% | 4 | 5.88% |
| 80144 | 3.13E-02 | 4 | 5.33% | 12 | 17.65% |
| 80235 | 4.88E-02 | 0 | 0.00% | 4 | 5.88% |
| 80336 | 4.88E-02 | 0 | 0.00% | 4 | 5.88% |
| 80778 | 4.88E-02 | 0 | 0.00% | 4 | 5.88% |
| 81539 | 1.02E-02 | 0 | 0.00% | 6 | 8.82% |
| 81617 | 4.88E-02 | 0 | 0.00% | 4 | 5.88% |
| 8216 | 4.88E-02 | 0 | 0.00% | 4 | 5.88% |
| 83439 | 1.02E-02 | 0 | 0.00% | 6 | 8.82% |
| 83607 | 4.88E-02 | 0 | 0.00% | 4 | 5.88% |
| 83903 | 1.36E-02 | 2 | 2.67% | 10 | 14.71% |
| 84069 | 2.25E-02 | 0 | 0.00% | 5 | 7.35% |
| 84283 | 4.88E-02 | 0 | 0.00% | 4 | 5.88% |
| 84467 | 3.92E-02 | 3 | 4.00% | 10 | 14.71% |
| 84619 | 4.88E-02 | 0 | 0.00% | 4 | 5.88% |
| 84631 | 1.37E-02 | 1 | 1.33% | 8 | 11.76% |
| 84698 | 1.37E-02 | 1 | 1.33% | 8 | 11.76% |
| 85442 | 3.92E-02 | 3 | 4.00% | 10 | 14.71% |
| 85461 | 2.56E-02 | 2 | 2.67% | 9 | 13.24% |
| 8659 | 2.25E-02 | 0 | 0.00% | 5 | 7.35% |
| 8671 | 4.88E-02 | 0 | 0.00% | 4 | 5.88% |
| 8736 | 1.87E-02 | 11 | 14.67% | 2 | 2.94% |
| 8786 | 4.88E-02 | 0 | 0.00% | 4 | 5.88% |
| 8839 | 4.88E-02 | 0 | 0.00% | 4 | 5.88% |
| 886 | 4.88E-02 | 0 | 0.00% | 4 | 5.88% |
| 8863 | 4.88E-02 | 0 | 0.00% | 4 | 5.88% |
| 8874 | 2.25E-02 | 0 | 0.00% | 5 | 7.35% |
| 8925 | 4.74E-02 | 2 | 2.67% | 8 | 11.76% |
| 8943 | 1.02E-02 | 0 | 0.00% | 6 | 8.82% |
| 8986 | 2.25E-02 | 0 | 0.00% | 5 | 7.35% |
| 89890 | 4.88E-02 | 0 | 0.00% | 4 | 5.88% |
| 90362 | 4.88E-02 | 0 | 0.00% | 4 | 5.88% |
| 9119 | 4.88E-02 | 0 | 0.00% | 4 | 5.88% |
| 91289 | 2.73E-02 | 1 | 1.33% | 7 | 10.29% |
| 9138 | 2.25E-02 | 0 | 0.00% | 5 | 7.35% |
| 91748 | 4.74E-02 | 2 | 2.67% | 8 | 11.76% |
| 91862 | 4.88E-02 | 0 | 0.00% | 4 | 5.88% |
| 9205 | 4.88E-02 | 0 | 0.00% | 4 | 5.88% |
| 9356 | 4.88E-02 | 0 | 0.00% | 4 | 5.88% |
| 94030 | 4.88E-02 | 0 | 0.00% | 4 | 5.88% |
| 9518 | 1.02E-02 | 0 | 0.00% | 6 | 8.82% |
| 9658 | 1.02E-02 | 0 | 0.00% | 6 | 8.82% |
| 9711 | 1.02E-02 | 0 | 0.00% | 6 | 8.82% |
| 9749 | 4.88E-02 | 0 | 0.00% | 4 | 5.88% |
| 9760 | 4.88E-02 | 0 | 0.00% | 4 | 5.88% |
| 9788 | 4.88E-02 | 0 | 0.00% | 4 | 5.88% |
| 9894 | 4.88E-02 | 0 | 0.00% | 4 | 5.88% |
| 9963 | 1.37E-02 | 1 | 1.33% | 8 | 11.76% |

^a^The number of samples with gene mutation in the high-risk/low-risk group ^b^The mutation frequencies in the high-risk/low-risk group.

**Supplementary Table S6**: Genes with mutations involving cell adhesion in the low-risk group.

| Gene symbol | Summary | Pathway |
| --- | --- | --- |
| ITGA3 | integrin alpha 3;The gene encodes a member of the integrin alpha chain family of proteins and function as cell surface adhesion molecules. | PI3K-Akt signaling/ECM-receptor interaction /Focal adhesion |
| ITGB4 | integrin beta 4; This gene encodes the integrin beta 4 subunit, a receptor for the laminins.Integrins mediate cell-matrix or cell-cell adhesion, and transduced signals that regulate gene expression and cell growth. | PI3K-Akt signaling/ECM-receptor interaction /Focal adhesion |
| LAMB1 | laminin, beta 1;Laminins, a family of extracellular matrix glycoproteins, are the major noncollagenous constituent of basement membranes. They have been implicated in a wide variety of biological processes including cell adhesion, differentiation, migration, signaling, neurite outgrowth and metastasis. | PI3K-Akt signaling/ECM-receptor interaction /Focal adhesion |
| COL5A3 | collagen, type V, alpha 3;This gene encodes an alpha chain for one of the low abundance fibrillar collagens. | PI3K-Akt signaling/ECM-receptor interaction /Focal adhesion |
| AGRN | agrin;This gene encodes one of several proteins that are critical in the development of the neuromuscular junction (NMJ), as identified in mouse knock-out studies. | ECM-receptor interaction |
| FGF13 | fibroblast growth factor 13;The protein encoded by this gene is a member of the fibroblast growth factor (FGF) family. FGF family members possess broad mitogenic and cell survival activities, and are involved in a variety of biological processes, including embryonic development, cell growth, morphogenesis, tissue repair, tumor growth, and invasion. | PI3K-Akt signaling |
| FLNA | filamin A, alpha; The encoded protein is involved in remodeling the cytoskeleton to effect changes in cell shape and migration. | Focal adhesion |
| FLNB | filamin B, beta;This gene encodes a member of the filamin family. The encoded protein interacts with glycoprotein Ib alpha as part of the process to repair vascular injuries. | Focal adhesion |
| HSPG2 | heparan sulfate proteoglycan 2; It is a major component of basement membranes, where it is involved in the stabilization of other molecules as well as being involved with glomerular permeability to macromolecules and cell adhesion. | ECM-receptor interaction |
| PIP5K1C | phosphatidylinositol-4-phosphate 5-kinase, type I, gamma;The encoded protein catalyzes phosphorylation of phosphatidylinositol 4-phosphate, producing phosphatidylinositol 4,5-bisphosphate. This enzyme is found at synapses and has been found to play roles in endocytosis and cell migration. | Focal adhesion |
| SGK3 | serum/glucocorticoid regulated kinase family member 3;This gene is a member of the Ser/Thr protein kinase family and encodes a phosphoprotein with a PX (phox homology) domain. The protein phosphorylates several target proteins and has a role in neutral amino acid transport and activation of potassium and chloride channels. | PI3K-Akt signaling |
| TSC2 | tuberous sclerosis 2 Its gene product is believed to be a tumor suppressor and is able to stimulate specific GTPases. | PI3K-Akt signaling |
| VWF | von Willebrand factor;This gene encodes a glycoprotein involved in hemostasis. | ECM-receptor interaction /Focal adhesion |

**Supplementary Table S7**: 1555 DEGs between the prognostic groups(Wilcoxon rank-sum test, FDR<5%) whose expression levels were negatively correlated with their methylation levels (Spearman correlation, FDR<5%).*P*-value was adjusted by Benjamini and Hochberg(FDR<5%).

| KEGG Pathway | P-value |
| --- | --- |
| Nicotinate and nicotinamide metabolism | 9.00E-04 |
| **Rap1 signaling pathway** | **1.31E-03** |
| Chemokine signaling pathway | 3.35E-04 |
| **PI3K-Akt signaling pathway** | **1.73E-03** |
| Vascular smooth muscle contraction | 1.95E-03 |
| **Cell adhesion molecules (CAMs)** | **2.13E-05** |
| Platelet activation | 4.65E-04 |
| Glutamatergic synapse | 6.99E-04 |
| Inflammatory mediator regulation of TRP channels | 1.90E-03 |

Note: Bold parts are pathways associated with 5-FU sensitivity.

**Supplementary Table S8:** 82 genes involved in 5-FU transport, metabolism and other downstream effects(such as DNA repair, apoptosis and cell cycle regulation),denoted as 5-FU activity-related genes, which were collected from a previous study[^1^](#_ENREF_1).

| Gene ID | Gene symbol | Function |
| --- | --- | --- |
| 85320 | ABCC11 | Transporters |
| 94160 | ABCC12 | Transporters |
| 10257 | ABCC4 | Transporters |
| 10057 | ABCC5 | Transporters |
| 50808 | AK3 | Metabolism (pyrimidine) |
| 10840 | ALDH1L1 | Metabolism (folate) |
| 275 | AMT | Metabolism (folate) |
| 471 | ATIC | Metabolism (folate) |
| 472 | ATM | DNArepair/apoptosis/cell cycle regulation |
| 545 | ATR | DNArepair/apoptosis/cell cycle regulation |
| 596 | BCL2 | DNArepair/apoptosis/cell cycle regulation |
| 124583 | CANT1 | Metabolism (pyrimidine) |
| 978 | CDA | Metabolism (pyrimidine) |
| 1066 | CES1 | Metabolism (pyrimidine) |
| 8824 | CES2 | Metabolism (pyrimidine) |
| 1111 | CHEK1 | DNArepair/apoptosis/cell cycle regulation |
| 11200 | CHEK2 | DNArepair/apoptosis/cell cycle regulation |
| 56474 | CTPS2 | Metabolism (pyrimidine) |
| 1719 | DHFR | Metabolism (folate) |
| 1806 | DPYD | Metabolism (pyrimidine) |
| 1807 | DPYS | Metabolism (pyrimidine) |
| 1841 | DTYMK | Metabolism (pyrimidine) |
| 1854 | DUT | Metabolism (pyrimidine) |
| 953 | ENTPD1 | Metabolism (pyrimidine) |
| 956 | ENTPD3 | Metabolism (pyrimidine) |
| 9583 | ENTPD4 | Metabolism (pyrimidine) |
| 957 | ENTPD5 | Metabolism (pyrimidine) |
| 955 | ENTPD6 | Metabolism (pyrimidine) |
| 9156 | EXO1 | DNArepair/apoptosis/cell cycle regulation |
| 10841 | FTCD | Metabolism (folate) |
| 2618 | GART | Metabolism (folate) |
| 3146 | HMGB1 | DNArepair/apoptosis/cell cycle regulation |
| 3704 | ITPA | Metabolism (pyrimidine) |
| 4292 | MLH1 | DNArepair/apoptosis/cell cycle regulation |
| 4436 | MSH2 | DNArepair/apoptosis/cell cycle regulation |
| 2956 | MSH6 | DNArepair/apoptosis/cell cycle regulation |
| 123263 | MTFMT | Metabolism (folate) |
| 4522 | MTHFD1 | Metabolism (folate) |
| 25902 | MTHFD1L | Metabolism (folate) |
| 10797 | MTHFD2 | Metabolism (folate) |
| 4524 | MTHFR | Metabolism (folate) |
| 10588 | MTHFS | Metabolism (folate) |
| 4548 | MTR | Metabolism (folate) |
| 4830 | NME1 | Metabolism (pyrimidine) |
| 4831 | NME2 | Metabolism (pyrimidine) |
| 4832 | NME3 | Metabolism (pyrimidine) |
| 4833 | NME4 | Metabolism (pyrimidine) |
| 8382 | NME5 | Metabolism (pyrimidine) |
| 10201 | NME6 | Metabolism (pyrimidine) |
| 29922 | NME7 | Metabolism (pyrimidine) |
| 401840 | NP | Metabolism (pyrimidine) |
| 30833 | NT5C | Metabolism (pyrimidine) |
| 84618 | NT5C1A | Metabolism (pyrimidine) |
| 93034 | NT5C1B | Metabolism (pyrimidine) |
| 22978 | NT5C2 | Metabolism (pyrimidine) |
| 51251 | NT5C3 | Metabolism (pyrimidine) |
| 4907 | NT5E | Metabolism (pyrimidine) |
| 56953 | NT5M | Metabolism (pyrimidine) |
| 318 | NUDT2 | Metabolism (pyrimidine) |
| 142 | PARP1 | DNArepair/apoptosis/cell cycle regulation |
| 10038 | PARP2 | DNArepair/apoptosis/cell cycle regulation |
| 5395 | PMS2 | DNArepair/apoptosis/cell cycle regulation |
| 87178 | PNPT1 | Metabolism (pyrimidine) |
| 6240 | RRM1 | Metabolism (pyrimidine) |
| 6241 | RRM2 | Metabolism (pyrimidine) |
| 50484 | RRM2B | Metabolism (pyrimidine) |
| 6470 | SHMT1 | Metabolism (folate) |
| 6472 | SHMT2 | Metabolism (folate) |
| 9154 | SLC28A1 | Transporters |
| 9153 | SLC28A2 | Transporters |
| 64078 | SLC28A3 | Transporters |
| 2030 | SLC29A1 | Transporters |
| 3177 | SLC29A2 | Transporters |
| 55315 | SLC29A3 | Transporters |
| 222962 | SLC29A4 | Transporters |
| 23583 | SMUG1 | DNArepair/apoptosis/cell cycle regulation |
| 7083 | TK1 | Metabolism (pyrimidine) |
| 7084 | TK2 | Metabolism (pyrimidine) |
| 7157 | TP53 | DNArepair/apoptosis/cell cycle regulation |
| 7296 | TXNRD1 | Metabolism (pyrimidine) |
| 10587 | TXNRD2 | Metabolism (pyrimidine) |
| 1890 | TYMP | Metabolism (pyrimidine) |
| 7298 | TYMS | Transporters |
| 83549 | UCK1 | Metabolism (pyrimidine) |
| 7371 | UCK2 | Metabolism (pyrimidine) |
| 7372 | UMPS | Metabolism (pyrimidine) |
| 7374 | UNG | DNArepair/apoptosis/cell cycle regulation |
| 51733 | UPB1 | Metabolism (pyrimidine) |
| 7378 | UPP1 | Metabolism (pyrimidine) |
| 151531 | UPP2 | Metabolism (pyrimidine) |
| 7515 | XRCC1 | DNArepair/apoptosis/cell cycle regulation |

**Supplementary Table S9:**The frequencies of the CMS subtypes predicted by the SSP method in the high-risk/low-risk groups, respectively.

|  |  | predictedCMS.SSP | |  | nearestCMS.SSP | |  |
| --- | --- | --- | --- | --- | --- | --- | --- |
| GSE39582 |  | High-risk (n=104)^a^ | Low-risk (n=96) | Fisher-test P-value | High-risk (n=104) | Low-risk (n=96) | Fisher-test P-value |
|  | CMS1 | 7.69% | 13.54% | 2.48E-01 | 13.46% | 17.71% | 4.39E-01 |
|  | CMS2 | 0.00% | 0.00% | - | 0.00% | 0.00% | - |
|  | CMS3 | 46.15% | **66.67%** | **4.33E-03** | 64.42% | **80.21%** | **1.77E-02** |
|  | CMS4 | **17.31%** | 1.04% | **4.57E-05** | **22.12%** | 2.08% | **8.52E-06** |
|  | NA | 28.85% | 18.75% | - | 0.00% | 0.00% | - |
| GSE14333 |  | High-risk (n=53) | Low-risk (n=32) | Fisher-test P-value | High-risk (n=53) | Low-risk (n=32) | Fisher-test P-value |
|  | CMS1 | 22.64% | 9.38% | 1.50E-01 | 33.96% | 15.63% | 2.59E-01 |
|  | CMS2 | 24.53% | **53.13%** | **1.02E-02** | 37.74% | **62.50%** | **4.28E-02** |
|  | CMS3 | 7.55% | 15.63% | 2.87E-01 | 11.32% | 18.75% | 3.55E-01 |
|  | CMS4 | **18.87%** | 3.13% | **4.63E-02** | **20.75%** | 3.13% | **2.63E-02** |
|  | NA | 32.08% | 18.75% | - | 0.00% | 0.00% | - |
| TCGA |  | High-risk (n=91) | Low-risk (n=93) | Fisher-test P-value | High-risk (n=91) | Low-risk (n=93) | Fisher-test P-value |
|  | CMS1 | 1.10% | 1.08% | 1.00E+00 | 5.49% | 16.13% | - |
|  | CMS2 | 2.20% | **22.58%** | **2.33E-05** | 9.89% | **35.48%** | **3.93E-05** |
|  | CMS3 | 13.19% | 18.28% | 4.20E-01 | 17.58% | 25.81% | 2.12E-01 |
|  | CMS4 | **45.05%** | 4.30% | **2.46E-11** | **67.03%** | 22.58% | **1.57E-09** |
|  | NA | 38.46% | 53.76% | - | 0.00% | 0.00% | - |

^a^The number of high-risk/low-risk patients predicted by the REO-based signature.

**Supplementary Table S10**: The comparisons between the study reported by Guinney et al and our work.

|  | Guinney et al | Our study |
| --- | --- | --- |
| The main significance of the study | development of a methodological gold standard for the taxonomy of CRC | identification of a REO-based prognostic signature for stage II-III CRC patients treated with 5-FU-based therapy |
| Molecular type of the classifier | mRNA | mRNA |
| Consensual description of CRC heterogeneity | Yes | No |
| Necessary for normalization procedures | Yes | No |
| Inflluence of batch effects | the classifier is sensitive to batch effects | the REO-based signature is largely free of batch effects |
| Evaluation of chemotherapy efficacy | the biological features of the CMS groups provided no information about which group could benefit from specific chemotherapy | the REOs-based signature could distinguish stage II-III CRC patients who are more likely to benefit from 5-FU-based therapy |

**References**

1 Tan WL, Bhattacharya B, Loh M, Balasubramanian I, Akram M, Dong D, et al. Low cytosine triphosphate synthase 2 expression renders resistance to 5-fluorouracil in colorectal cancer. Cancer Biol Ther 2011; 11:599-608.

2 Huehls AM, Huntoon CJ, Joshi PM, Baehr CA, Wagner JM, Wang X, et al. Genomically Incorporated 5-Fluorouracil that Escapes UNG-Initiated Base Excision Repair Blocks DNA Replication and Activates Homologous Recombination. Mol Pharmacol 2015.

3 Martino-Echarri E, Henderson BR, Brocardo MG. Targeting the DNA replication checkpoint by pharmacologic inhibition of Chk1 kinase: a strategy to sensitize APC mutant colon cancer cells to 5-fluorouracil chemotherapy. Oncotarget 2014; 5:9889-9900.

4 Longley DB, Harkin DP, Johnston PG. 5-fluorouracil: mechanisms of action and clinical strategies. Nat Rev Cancer 2003; 3:330-338.

5 Heffler M, Golubovskaya VM, Dunn KM, Cance W. Focal adhesion kinase autophosphorylation inhibition decreases colon cancer cell growth and enhances the efficacy of chemotherapy. Cancer Biol Ther 2013; 14:761-772.

6 Naba A, Clauser KR, Whittaker CA, Carr SA, Tanabe KK, Hynes RO. Extracellular matrix signatures of human primary metastatic colon cancers and their metastases to liver. BMC Cancer 2014; 14:518.

7 Shigeta K, Ishii Y, Hasegawa H, Okabayashi K, Kitagawa Y. Evaluation of 5-fluorouracil metabolic enzymes as predictors of response to adjuvant chemotherapy outcomes in patients with stage II/III colorectal cancer: a decision-curve analysis. World J Surg 2014; 38:3248-3256.

8 Zollner N. Purine and pyrimidine metabolism. Proc Nutr Soc 1982; 41:329-342.

9 Seetharam R, Sood A, Goel S. Oxaliplatin: pre-clinical perspectives on the mechanisms of action, response and resistance. Ecancermedicalscience 2009; 3:153.

10 Fu L, Lee CC. The circadian clock: pacemaker and tumour suppressor. Nat Rev Cancer 2003; 3:350-361.

11 Zhang B, Chen X, Bae S, Singh K, Washington MK, Datta PK. Loss of Smad4 in colorectal cancer induces resistance to 5-fluorouracil through activating Akt pathway. Br J Cancer 2014; 110:946-957.

12 Chen Q, Li W, Wan Y, Xia X, Wu Q, Chen Y, et al. Amplified in breast cancer 1 enhances human cholangiocarcinoma growth and chemoresistance by simultaneous activation of Akt and Nrf2 pathways. Hepatology 2012; 55:1820-1829.

13 Buhrmann C, Shayan P, Kraehe P, Popper B, Goel A, Shakibaei M. Resveratrol induces chemosensitization to 5-fluorouracil through up-regulation of intercellular junctions, Epithelial-to-mesenchymal transition and apoptosis in colorectal cancer. Biochem Pharmacol 2015; 98:51-68.

14 Edelman GM. Cell adhesion molecules. Science 1983; 219:450-457.

15 Downward J. Targeting RAS signalling pathways in cancer therapy. Nat Rev Cancer 2003; 3:11-22.

16 Sugita K, Morohashi Y, Sato C. [Key to efficient patient education on diet therapy]. Kurinikaru Sutadi 1989; 10:808-809.

17 Zha Y, Gan P, Yao Q, Ran FM, Tan J. Downregulation of Rap1 promotes 5-fluorouracil-induced apoptosis in hepatocellular carcinoma cell line HepG2. Oncol Rep 2014; 31:1691-1698.
