# Supplementary material for: Multi-omics landscapes of colorectal cancer subtypes discriminated by an individualized prognostic signature for 5-fluorouracil-based chemotherapy
Source: Oncogenesis. 2016 Jul 18;5(7):e242–. doi: 10.1038/oncsis.2016.51 (PMC5399173; doi:10.1038/oncsis.2016.51)
Supplement: Supplementary Information [file oncsis201651x2.pdf]

Supplementary Data1: The prevalence of copy number alterations for the 12 pathways associated with 5-FU sensitivity among the 91 high-risk samples.

Pathway	Cell cycle	PI3K-Akt signaling pathway	Ras signaling pathway	Focal adhesion	Rap1 signaling pathway	Pyrimidine metabolism	DNA replication	Mismatch repair	Base excision repair	Nucleotide excision repair	Cell adhesion molecules (CAMs)	ECM-receptor interaction
No. of Sample	80	79	78	78	74	74	65	61	49	62	23	0
Frequency	87.91%	86.81%	85.71%	85.71%	81.32%	81.32%	71.43%	67.03%	53.85%	68.13%	25.28%	0.00%
No. of pathway	17	29	23	13	23	5	2	3	3	3	1	0
TCGA-A6-4105	7	14	11	6	14	0	0	1	0	2	1	0
TCGA-F4-6463	4	4	3	1	3	0	1	0	1	0	0	0
TCGA-AA-3675	1	1	2	1	3	0	0	0	0	0	0	0
TCGA-DM-A1D7	8	15	10	8	11	3	0	1	1	1	0	0
TCGA-DM-A28C	0	0	0	0	0	0	0	0	0	0	0	0
TCGA-G4-6310	9	11	11	7	12	4	2	2	1	1	0	0
TCGA-DM-A1D6	9	9	10	5	12	2	1	2	0	1	1	0
TCGA-F4-6459	8	12	9	7	10	3	2	2	1	1	0	0
TCGA-AD-6899	10	13	12	7	14	2	2	3	1	2	0	0
TCGA-A6-2675	10	13	13	8	11	4	2	2	1	2	0	0
TCGA-DM-A1D9	9	14	12	7	13	4	2	3	1	2	0	0
TCGA-A6-5664	2	1	0	1	0	3	0	0	0	0	0	0
TCGA-A6-A5ZU	8	12	13	6	14	4	2	2	2	2	0	0
TCGA-D5-6529	9	6	7	4	6	1	1	0	1	1	0	0
TCGA-A6-6649	5	13	10	7	12	3	2	3	1	2	0	0
TCGA-A6-A566	2	3	4	4	5	2	1	2	0	1	0	0
TCGA-D5-6536	6	9	12	6	14	4	1	2	1	2	0	0
TCGA-CA-5796	1	2	4	3	5	2	1	2	0	1	0	0
TCGA-A6-A56B	12	16	16	8	17	5	1	2	1	1	1	0
TCGA-CM-6172	7	6	7	5	8	3	2	2	1	1	0	0
TCGA-A6-6654	13	24	17	10	21	5	2	3	1	2	1	0
TCGA-F4-6854	6	12	11	9	12	3	1	3	0	2	0	0
TCGA-CK-4951	0	0	0	0	0	3	0	0	0	0	0	0
TCGA-G4-6321	0	0	0	0	0	3	0	0	0	0	0	0
TCGA-AZ-6605	12	17	16	8	18	2	2	3	1	2	1	0
TCGA-AA-3489	5	3	8	5	8	2	1	2	0	1	0	0
TCGA-CM-5348	6	10	8	6	9	3	2	2	1	1	0	0
TCGA-CM-6674	0	0	0	0	0	3	0	0	0	0	0	0
TCGA-F4-6460	14	23	19	11	21	4	2	3	0	3	1	0
TCGA-D5-6928	2	2	2	2	3	3	0	0	0	0	0	0
TCGA-D5-6932	13	22	16	8	16	1	1	1	1	2	1	0
TCGA-CM-5860	8	13	9	7	10	3	2	2	1	1	0	0
TCGA-D5-5537	3	6	4	3	8	1	0	1	0	1	0	0
TCGA-G4-6323	3	5	6	6	8	2	1	2	0	1	0	0
TCGA-WS-AB45	4	5	5	3	5	2	2	2	1	1	0	0
TCGA-F4-6461	4	4	3	1	2	1	0	0	1	0	0	0
TCGA-CM-5344	9	14	16	7	16	4	1	2	1	2	1	0
TCGA-CM-6168	5	6	7	4	8	2	2	2	1	1	0	0
TCGA-CM-4751	7	5	7	4	5	2	2	2	1	1	0	0
TCGA-F4-6807	12	13	12	4	11	2	1	0	2	0	1	0
TCGA-CM-6165	5	3	8	5	8	2	1	2	0	1	0	0
TCGA-CM-4743	0	0	0	0	0	3	0	0	0	0	0	0
TCGA-A6-6782	9	12	11	8	11	3	2	3	1	2	0	0
TCGA-AY-6196	9	18	15	10	17	3	2	3	0	3	0	0
TCGA-AA-A01P	0	0	0	0	0	3	0	0	0	0	0	0
TCGA-D5-6924	12	19	14	8	16	2	2	3	1	2	1	0
TCGA-G4-6293	2	3	6	4	8	2	1	2	0	1	0	0
TCGA-D5-6534	8	11	12	6	13	4	2	2	2	2	0	0
TCGA-AZ-4315	0	0	0	0	0	3	0	0	0	0	0	0
TCGA-DM-A282	3	1	2	1	3	3	0	0	0	0	0	0
TCGA-CM-6680	4	0	2	1	0	3	0	0	0	0	0	0
TCGA-F4-6805	6	4	2	2	2	0	1	0	1	0	0	0
TCGA-G4-6322	8	10	11	4	9	1	0	0	1	0	1	0
TCGA-A6-6137	5	5	4	1	4	0	1	0	1	0	0	0
TCGA-AA-3511	10	16	16	9	16	4	2	3	2	3	0	0
TCGA-A6-5660	13	15	14	8	16	4	2	2	0	1	1	0
TCGA-NH-A50V	4	6	6	4	8	0	0	1	0	1	0	0
TCGA-F4-6855	10	10	13	6	13	3	1	2	0	1	1	0
TCGA-D5-6926	11	15	14	8	12	5	2	2	2	1	0	0
TCGA-A6-6141	0	0	0	0	0	0	0	0	0	0	0	0
TCGA-A6-6651	9	15	14	6	15	1	0	1	0	2	1	0
TCGA-G4-6627	8	9	8	5	9	2	2	2	1	1	0	0
TCGA-G4-6299	6	4	5	4	7	2	2	2	0	1	0	0
TCGA-F4-6703	3	1	2	2	0	0	0	0	0	0	0	0

TCGA-AM-5821	8	11	6	2	5	0	1	0	1	0	1	0
TCGA-F4-6704	11	15	15	8	17	4	1	2	0	1	1	0
TCGA-DM-A0X9	5	5	3	2	3	0	1	0	1	0	0	0
TCGA-CM-6167	6	8	8	4	9	2	2	2	1	1	0	0
TCGA-G4-6298	10	10	10	5	12	3	2	2	0	1	1	0
TCGA-NH-A6GA	8	9	10	5	12	3	2	2	0	2	1	0
TCGA-D5-5541	7	10	8	6	9	3	2	2	1	1	0	0
TCGA-CA-6716	10	16	15	7	15	1	0	1	1	2	1	0
TCGA-A6-A565	1	2	4	3	5	2	1	2	0	1	0	0
TCGA-CM-6679	14	20	18	9	19	3	2	3	2	2	1	0
TCGA-A6-2685	10	12	11	5	13	3	2	2	0	1	1	0
TCGA-G4-6311	7	10	8	6	9	3	2	2	1	1	0	0
TCGA-D5-5539	6	12	9	7	11	3	1	2	0	1	0	0
TCGA-CM-6162	0	0	0	0	0	0	0	0	0	0	0	0
TCGA-CM-5349	8	10	4	5	6	2	1	0	1	0	0	0
TCGA-AA-3496	6	7	8	4	9	2	2	2	1	1	0	0
TCGA-D5-6539	7	10	7	8	11	3	1	2	0	1	0	0
TCGA-CM-6169	14	26	15	8	15	3	1	1	2	1	1	0
TCGA-AZ-5403	11	13	14	6	12	3	2	2	2	1	0	0
TCGA-G4-6302	3	3	1	0	0	0	1	0	1	0	0	0
TCGA-CA-6719	10	15	12	7	14	5	2	2	2	1	0	0
TCGA-D5-6922	11	15	14	8	12	4	2	2	2	1	0	0
TCGA-CA-6717	0	0	0	0	0	0	0	0	0	0	0	0
TCGA-D5-6541	6	4	1	1	0	0	1	0	1	0	0	0
TCGA-A6-6781	0	0	0	0	0	0	0	0	0	0	0	0
TCGA-DM-A28G	3	2	0	0	0	0	1	0	0	0	0	0
TCGA-AY-A69D	10	15	13	6	14	2	0	1	1	1	1	0

Supplementary Data2: The prevalence of mutations for the 12 pathways associated with 5-FU sensitivity among the 68 low-risk samples.

Pathway	ECM-receptor interaction	Focal adhesion	PI3K-Akt signaling pathway	Cell adhesion molecules (CAMs)	DNA replication	Pyrimidine metabolism	Base excision repair	Rap1 signaling pathway	Ras signaling pathway	Nucleotide excision repair	Mismatch repair	Cell cycle
No. of Sample	31	30	27	17	12	11	11	11	10	9	9	8
Frequency	45.59%	44.12%	39.71%	25.00%	17.65%	16.18%	16.18%	16.18%	14.71%	13.24%	13.24%	11.76%
No. of pathway gene	7	8	8	3	2	2	2	2	3	1	1	2
TCGA-A6-5657	0	0	0	0	0	0	0	0	0	0	0	0
TCGA-A6-5661	2	3	3	2	0	0	0	0	1	0	0	1
TCGA-A6-5665	1	2	1	1	2	1	1	1	1	1	1	1
TCGA-A6-5666	0	0	0	0	0	0	0	0	0	0	0	0
TCGA-A6-5667	1	1	1	1	0	0	0	0	0	0	0	0
TCGA-A6-6140	0	0	0	0	0	1	0	0	0	0	0	0
TCGA-A6-6650	0	0	0	0	0	0	0	0	0	0	0	0
TCGA-A6-6780	2	2	1	1	1	2	2	0	0	1	1	0
TCGA-AA-3492	2	1	3	0	1	1	0	1	2	0	0	2
TCGA-AA-3655	0	0	0	0	0	0	0	0	0	0	0	0
TCGA-AA-3660	0	0	0	0	0	0	0	0	0	0	0	0
TCGA-AA-3663	1	3	1	1	2	1	1	1	1	1	1	1
TCGA-AA-3697	1	2	1	1	0	0	0	1	0	0	0	0
TCGA-AA-3712	1	1	2	1	1	0	0	1	0	0	0	1
TCGA-AD-6888	0	0	0	0	0	0	0	0	0	0	0	0
TCGA-AD-6889	3	4	2	0	1	0	0	0	1	0	0	2
TCGA-AU-3779	0	0	0	1	0	0	0	0	0	0	0	0
TCGA-AY-6197	5	5	4	0	1	2	2	0	0	1	1	0
TCGA-AY-6386	1	0	0	0	0	0	0	1	0	0	0	0
TCGA-AZ-4615	2	1	2	1	0	0	1	0	0	0	0	0
TCGA-AZ-6598	1	2	1	1	2	1	1	0	0	1	1	1
TCGA-AZ-6601	4	3	3	0	0	0	0	0	0	0	0	0
TCGA-CA-5797	0	0	0	0	0	0	0	0	0	0	0	0
TCGA-CA-6718	1	2	1	0	0	0	0	0	0	0	0	0
TCGA-CK-4950	1	0	0	1	0	0	0	0	0	0	0	0
TCGA-CK-4952	0	0	0	0	0	0	0	0	1	0	0	0
TCGA-CK-5913	2	3	2	0	1	1	1	0	0	1	1	0
TCGA-CK-5914	0	0	0	0	0	0	0	0	1	0	0	0
TCGA-CM-5861	2	3	2	0	1	1	1	0	0	1	1	0
TCGA-CM-5863	0	0	0	0	0	0	0	0	0	0	0	0
TCGA-CM-6164	0	0	0	0	0	0	0	0	0	0	0	0
TCGA-CM-6677	1	1	1	0	0	0	0	0	0	0	0	0
TCGA-D5-5538	0	0	0	0	0	0	0	0	0	0	0	0
TCGA-D5-5540	1	0	0	0	0	0	0	0	0	0	0	0
TCGA-D5-6531	0	0	0	0	0	0	0	1	0	0	0	0
TCGA-D5-6532	0	0	0	0	0	0	0	0	0	0	0	0
TCGA-D5-6533	0	0	0	0	0	0	0	0	0	0	0	0
TCGA-D5-6535	0	0	0	0	0	0	0	0	0	0	0	0
TCGA-D5-6537	0	0	0	0	0	0	0	1	1	0	0	0
TCGA-D5-6538	0	1	0	0	0	0	0	0	0	0	0	0
TCGA-D5-6920	0	1	0	1	0	0	0	0	0	0	0	0
TCGA-D5-6927	1	0	0	0	1	1	1	0	0	1	1	0
TCGA-D5-6930	4	2	3	1	0	0	0	1	0	0	0	0
TCGA-D5-6931	1	1	1	1	0	0	0	0	0	0	0	0
TCGA-DM-A0XD	0	0	0	0	0	0	0	0	0	0	0	0
TCGA-DM-A0XF	0	0	0	0	0	0	0	0	0	0	0	0
TCGA-DM-A1D0	0	0	0	0	0	0	0	0	0	0	0	0
TCGA-DM-A1D4	0	0	0	0	0	0	0	0	0	0	0	0
TCGA-DM-A1DA	1	1	1	0	0	0	0	0	0	0	0	0
TCGA-DM-A1DB	1	1	1	1	0	0	0	0	0	0	0	0
TCGA-DM-A1HA	0	0	0	0	0	0	0	0	0	0	0	0
TCGA-DM-A28E	0	0	0	0	0	0	0	0	0	0	0	0
TCGA-DM-A28F	0	0	0	0	0	0	1	0	0	0	0	0
TCGA-DM-A28H	0	0	0	0	0	0	0	0	0	0	0	0
TCGA-DM-A28K	0	0	0	0	0	0	0	0	0	0	0	0
TCGA-DM-A28M	0	0	0	0	0	0	0	0	0	0	0	0
TCGA-F4-6570	4	3	3	0	0	0	0	2	1	0	0	0
TCGA-G4-6295	0	0	0	0	0	0	0	0	0	0	0	0
TCGA-G4-6304	0	0	0	0	0	0	0	0	0	0	0	0

TCGA-G4-6306	0	1	0	0	0	0	0	0	0	0	0	0
TCGA-G4-6307	0	0	0	0	0	0	0	0	0	0	0	0
TCGA-G4-6309	2	2	2	1	0	0	0	0	0	0	0	0
TCGA-G4-6317	0	0	0	0	0	0	0	0	0	0	0	0
TCGA-G4-6320	2	2	2	0	0	0	0	0	0	0	0	0
TCGA-G4-6586	3	3	2	0	0	0	0	0	0	0	0	0
TCGA-G4-6588	2	5	3	1	2	2	1	1	2	1	1	2
TCGA-G4-6625	1	1	1	0	0	0	0	0	0	0	0	0
TCGA-G4-6626	0	0	0	0	0	0	0	0	0	0	0	0
